# Distinct patterns of innate immune activation by clinical isolates of respiratory syncytial virus

**DOI:** 10.1371/journal.pone.0184318

**Published:** 2017-09-06

**Authors:** Ruth Levitz, Yajing Gao, Igor Dozmorov, Ran Song, Edward K. Wakeland, Jeffrey S. Kahn

**Affiliations:** 1 Department of Pediatrics, University of Texas Southwestern Medical Center, Dallas, Texas, United States of America; 2 Department of Immunology, University of Texas Southwestern Medical Center, Dallas, Texas, United States of America; 3 Department of Microbiology, University of Texas Southwestern Medical Center, Dallas, Texas, United States of America; University of Georgia, UNITED STATES

## Abstract

Respiratory syncytial virus (RSV) is a major respiratory pathogen of infants and young children. Multiple strains of both subgroup A and B viruses circulate during each seasonal epidemic. Genetic heterogeneity among RSV genomes, in large part due to the error prone RNA-dependent, RNA polymerase, could mediate variations in pathogenicity. We evaluated clinical strains of RSV for their ability to induce the innate immune response. Subgroup B viruses were used to infect human pulmonary epithelial cells (A549) and primary monocyte-derived human macrophages (MDM) from a variety of donors. Secretions of IL-6 and CCL5 (RANTES) from infected cells were measured following infection. Host and viral transcriptome expression were assessed using RNA-SEQ technology and the genomic sequences of several clinical isolates were determined. There were dramatic differences in the induction of IL-6 and CCL5 in both A549 cells and MDM infected with a variety of clinical isolates of RSV. Transcriptome analyses revealed that the pattern of innate immune activation in MDM was virus-specific and host-specific. Specifically, viruses that induced high levels of secreted IL-6 and CCL5 tended to induce cellular innate immune pathways whereas viruses that induced relatively low level of IL-6 or CCL5 did not induce or suppressed innate immune gene expression. Activation of the host innate immune response mapped to variations in the *RSV G* gene and the *M2-1* gene. Viral transcriptome data indicated that there was a gradient of transcription across the RSV genome though in some strains, *RSV G* was the expressed in the highest amounts at late times post-infection. Clinical strains of RSV differ in cytokine/chemokine induction and in induction and suppression of host genes expression suggesting that these viruses may have inherent differences in virulence potential. Identification of the genetic elements responsible for these differences may lead to novel approaches to antiviral agents and vaccines.

## Introduction

Respiratory syncytial virus (RSV) is a major respiratory pathogen of infants and children worldwide. RSV is the leading cause of hospitalization of young children in the United States accounting for ~80,000 hospitalizations each year[1]. Globally, RSV is the most common cause of acute lower respiratory tract infection responsible for significant mortality in children less than 5 years of age (up to 199,000 deaths per year), mostly in developing countries [2]. RSV is responsible for ~50% of all pneumonias in infancy[3]. The virus is ubiquitous—by the age of 2 years, nearly every child has exposed to and infected with RSV[4].

The epidemiology of RSV is complex and dynamic. RSV strains can be classified, based on serological [5] or genetic methods [6], into 2 subgroups, A or B. Both subgroup A and B viruses circulate during seasonal epidemics (typically the late fall, winter and early spring in temperate climates) and unlike influenza virus pandemics, RSV strains may vary from location to location in any given RSV season [6]. Likewise, strains identified in one location may be similar to strains from vastly different geographic locations identified in different years [7].

The pathogenesis of RSV is not fully defined. Several animal models, including mice, cotton rats, and non-human primates, have been used to study RSV infection [8–10]. The vast majority of these investigations have used laboratory strains of RSV or recombinant virus derived from these laboratory strains. Many of these strains were isolated decades ago. For example, the reference A2 strain was isolated in Australia in 1961, and the passage histories of this strain, as with most laboratory strains of RSV, are not known or poorly documented. It is unclear whether these strains have adapted to cell culture systems or to animal models in which they are used, and in so doing, acquired mutations. Therefore, these strains may not be authentic representatives of feral strains of RSV. In fact, the reference A2 strain differs from several clinical isolates in its ability to replicate on primary human bronchial epithelial cells and induce interferon-inducible protein 10 and CCL5 suggesting that A2 alone may not be the ideal strain to study RSV pathogenesis [11]. For example, Line 19, a potential vaccine strain derived from a subgroup A isolate possesses biological features that differ from the reference A2 strain. This phenotype has been mapped to the RSV F gene [12]. Furthermore, wild-type RSV strains are highly polymorphic and are likely to contain variations that impact virulence. Stokes et al demonstrated that clinical isolates varied in their capacity to induce airway mucous production in mice [13]. In this study, 6 clinical subgroup A isolates, representing difference subgroup A clades and apparently chosen randomly, displayed distinct replication kinetics, IL-13 and gob-5 induction and mucin production in BALB/c mice. These studies, which relied, for the most part, on randomly chosen clinical strains, strongly suggest that a more thorough, focused and systematic investigation of clinical isolates of RSV may lead to the identification of strains with dramatic differences in phenotype, specifically, induction of innate immunity and inflammation and may lead to the identification of previously unrecognized viral virulence factors.

We have previously screened subgroup B isolates for their capacity to induce cytokines and chemokines, specifically IL-6 and CCL5 (RANTES), in A549 cells, a continuous human pulmonary epithelial cell line [14]. IL-6 is secreted by a variety of cell types including T cells and macrophages. In T cells and macrophages, IL-6 is secreted in response to infection and acts as a pro-inflammatory cytokine. IL-6 is required for protection against a variety of microbial pathogens; deficiency of this molecule results in impair innate and adaptive immune responses to viral infections [15]. CCL5, a chemokine, attracts immune and inflammatory cells to the site of infection and is secreted by a variety of immune cells, among them macrophages and non-immune cells such as epithelial cells [16]. We chose to study these 2 molecules because there is *in vitro*, animal and clinical data to support that these factors are induced during RSV infection and may play a role in the immune and inflammatory response to RSV [17–23]. We found that 2 isolates, NH1125B and NH1067B, differed in their ability to induce IL-6 and CCL5. The replication kinetics of the 2 viruses were essentially identical suggesting that the observed difference is cytokine and chemokine induction could not be explained by differences in the kinetics of viral replication.

Here, we have used comprehensive RNA-SEQ analyses to characterize both host cell and viral strain gene expression. Our results indicate that NH1125B and NH1067B differ significantly in their ability to control innate immune activation early in the viral infection cycle. This functional variation coincides with the presence of distinct features within the *G* gene, specifically a 60 base duplication and a single amino acid polymorphism and or a single amino acid polymorphism in the M2-1 protein.

## Results

### Magnitude of induction of IL-6 and CCL5 in A549 cells and primary monocyte-derived human macrophages (MDM) by clinical isolates of RSV is strain- and host-specific

**Fig 1:**
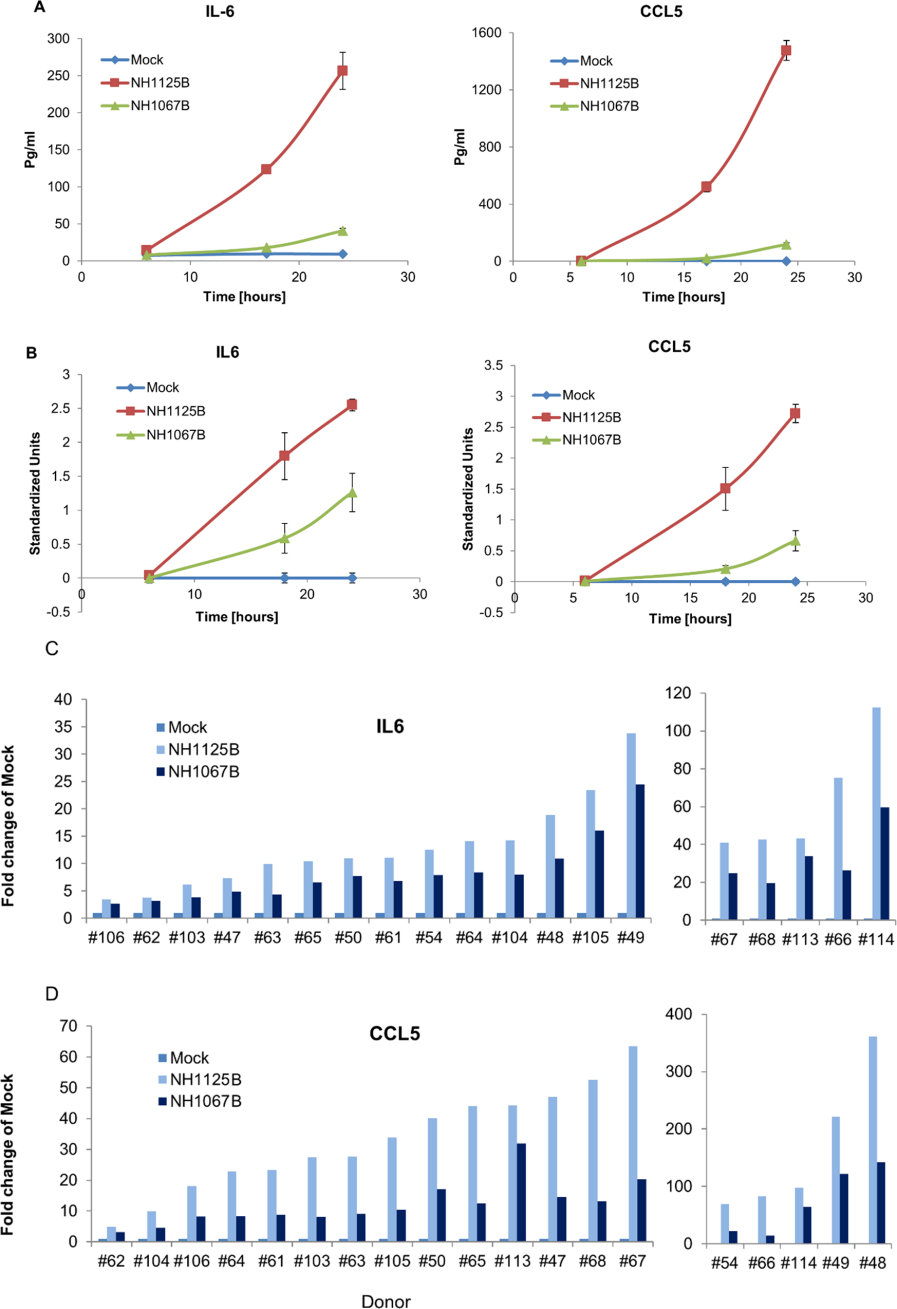
Induction of cytokines and chemokines by clinical isolates of RSV in A549 cells and primary monocyte-derived human macrophages (MDM). (A) A549 cells or (B) MDM (donor #66, 67 and 68) were infected with clinical isolates NH1125B or NH1067B. Mock infected cells were used as controls. At multiple time points post-infection, cell culture supernatant were collected and concentration of IL-6 and RANTES (CCL5) were measured by BioPlex assay. For the A549 cell experiments, biological triplicates were used and the error bars are displayed (for several time points, the error bars are too small to be seen in the figure). To make the MDM data from different donors compatible, we applied a standardization procedure with the average expression over all time points (for each donor) equal zero and the standard deviation equal 1. Data along ordinate axis is given in these standardized units. Nineteen donors (including donor #66, 67 and 68) were screened for secretion of IL-6 (C) and CCL5 (D) at 24 hours post infection using the methodologies described above.

We have previously demonstrated that clinical isolates of RSV differ in their capacity to induce cytokines and chemokines in a standard human respiratory epithelial cell line, A549 [14]. To determine whether this was the case during infection of monocyte-derived human macrophages (MDM), NH1125B and NH1067B were used to infect MDM (donor #66, 67 and 68). At several time points post infection, supernatants were collected and the concentrations of IL-6 and CCL5 determined by BioPlex assay (Fig 1A and 1B). These time points were chosen, in large part, because initial experiments indicated that at early times post infection (typically 8–18 hours) the most significant differences between the 2 viruses were observed. We included later times (up to 24 hours post infection) as subsequent experiments suggested that the induction patterns of NH1067B trended towards that observed for NH1125B. For A549 cells and MDM, the induction of IL-6 and CCL5 was greater during infection with NH1125B than NH1067B at both 18 and 24 hours post-infection. To explore whether this observation was specific to these 3 donors or an inherent property of the viral strains, we screened MDMs from an additional 16 donors for induction of IL-6 and CCL5 during viral infection. In each case, IL-6 and CCL5 induction was greater during infection with NH1125B than NH1067B (Fig 1C and 1D, respectively) indicating that there are likely viral-specific factors involved in the degree of cytokine and chemokine induction during infection.

**Fig 2:**
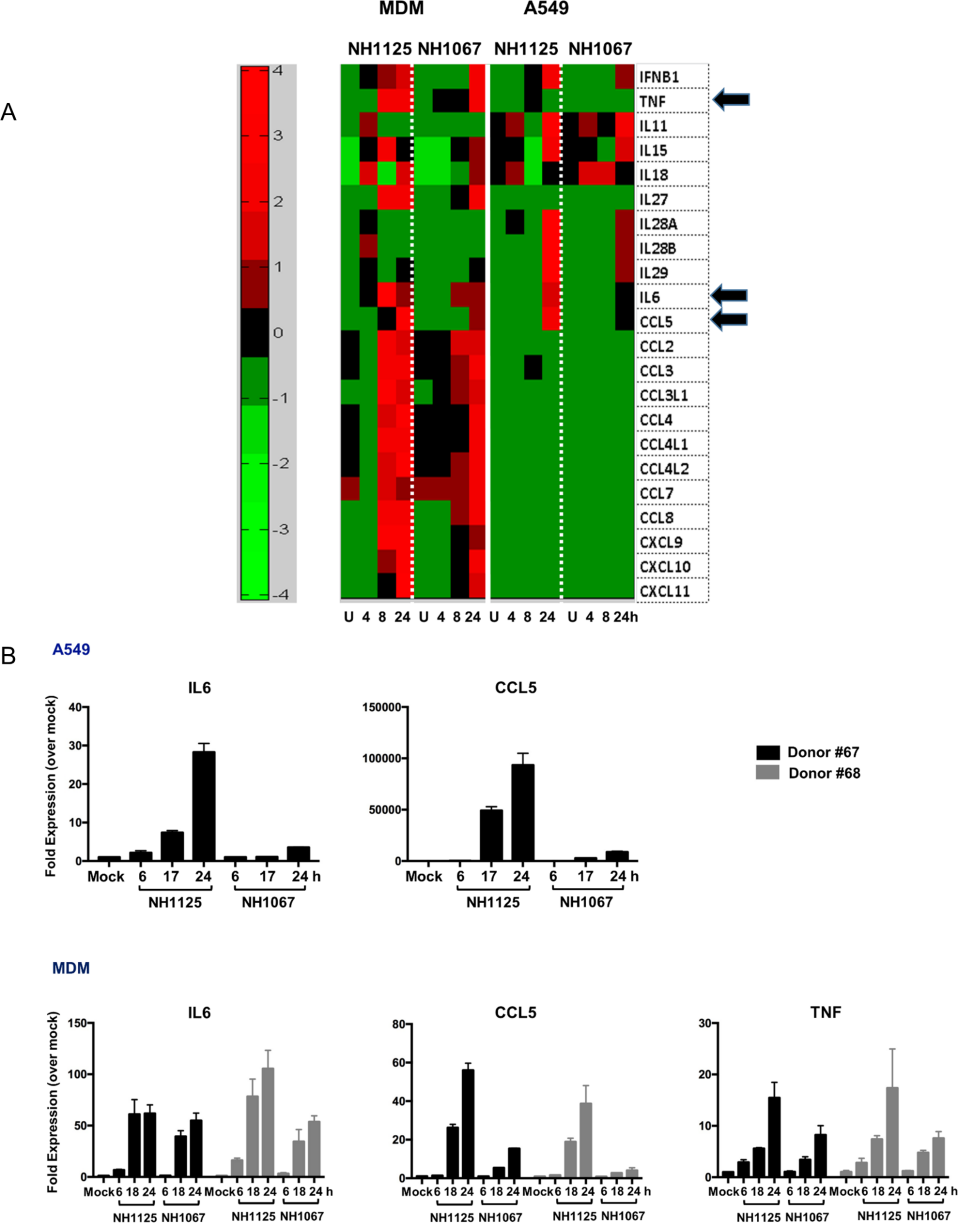
Transcriptome analyses of A549 cells and MDM during infection with clinical isolates of RSV. A549 cells and MDM (donor#64) were infected with NH1067B and NH1125B. RNA was extracted at the designated times post infection and gene expression was determined by RNA-SEQ (A) or quantitative RT-PCR (B). (A) Heat map illustrating changes in cytokine and chemokine transcription following infection. Individual genes are in rows and variations in expression are depicted using the color scale on the right of the figure. Each column is a time point (in hours) post infection. “U” represents mock infected cells. Each row represents a unique cytokine. Arrows point to transcription of IL-6, CCL5 and TNFα. (B) Quantitative RT-PCR of specific genes in virus infected A549 and MDMs. RNA was extracted from MDM from donor #67 and 68 at several times post infection (as described above) and gene expression was determined by quantitative RT-PCR. The Y-axis represents fold expression over mock infected cells and error bars are included for statistical perspectives.

These initial observations were broadened by the analysis of transcriptome sequence data obtained from virus-infected cells (Fig 2A) which demonstrates variations in the induction of multiple cytokines transcription in both infected A549 and MDM cells. Furthermore, MDM cells transcribe many more chemokines than A549 cells infected identically consistent with the well-established potency of the macrophage to induce inflammation during infections. The differences in the expression of IL-6, CCL5 and TNFα were confirmed using quantitative RT-PCR (Fig 2B). Consistent with our RNA-SEQ data, the induction of IL-6 and CCL5 in A549 cells was significantly greater in cells infected with NH1125B as compared to NH1067B (there was minimal induction of TNFα in A549 cells is response to either virus so this data was not included in the figure). NH1125B, as compared to NH1067B, induced IL-6 earlier during infection and induced greater levels of CCL5 and TNFα transcripts in the MDM of the 2 donors (#67 and #68) presented. Host transcriptome data for A549 cells and MDMs from these 2 donors supported the differences in the time of gene expression by the NH1125B and NH1067B (S1 Fig; all genes displayed are statistically significantly different vs. mock infected cells). Although the overall patterns of gene expression in cells infected with NH1125B and NH1067B were, in general similar, there were distinct differences in the kinetics of gene expression between the 2 viruses. Based on these data, the induction of several cellular pathways was deciphered (Panel A-I in S2 Fig) providing insights into the defined cellular responses to RSV infection. In all, the level of induction of cytokine and chemokine genes was dependent on the infecting virus strain, with NH1125B allowing significantly greater transcription levels of many innate immune response genes. The replication kinetics of NH1125B and NH1067B were essentially identical [14] indicating that some other intrinsic property of the viruses, other than replication dynamics, drives the innate immune response.

### Induction of IL-6 and CCL5 by clinical isolates of RSV is blocked by an RSV-neutralizing antibody

One of the risks in using virus isolated from clinical specimens is that prepared viral stocks (even if plaque-purified 3 times as is the case with all viruses used in this set of experiments) may contain undetectable viruses that may contribute to cytokine induction and therefore result in misleading data. To address this issue and to determine whether infection with RSV was necessary for induction of IL-6 and CCL5 in MDM, a virus neutralization experiment was performed using clinical isolate NH1125B (S3 Fig). Consistent with data presented in Fig 1, NH1125B induced IL-6 and CCL5 in MDM from 2 independent donors (#96 and 98). This induction was blocked by incubation of NH1125B with an RSV-neutralizing antibody (palivizumab, SYN) prior to infection (Panel A, B in S3 Fig). To establish that the RSV neutralizing antibody did not independently affect the induction of IL-6 or CCL5, MDM were stimulated with the TLR7/8 stimulator (R848) in the absence and presence of the RSV neutralizing antibody (using the same concentration as that used in the virus-neutralization experiment). The RSV-neutralizing antibody did not have any effect on the induction of IL-6 and CCL5 (Panel C, D in S3 Fig). This series of experiments indicated that the induction of IL-6 and CCL5 by NH1125B is virus specific, requires viral infection and was not due to some other factor (e.g. virus) in the viral stock preparation.

### Activation of host gene expression differs during infection with NH1125B and NH1067B

**Fig 3:**
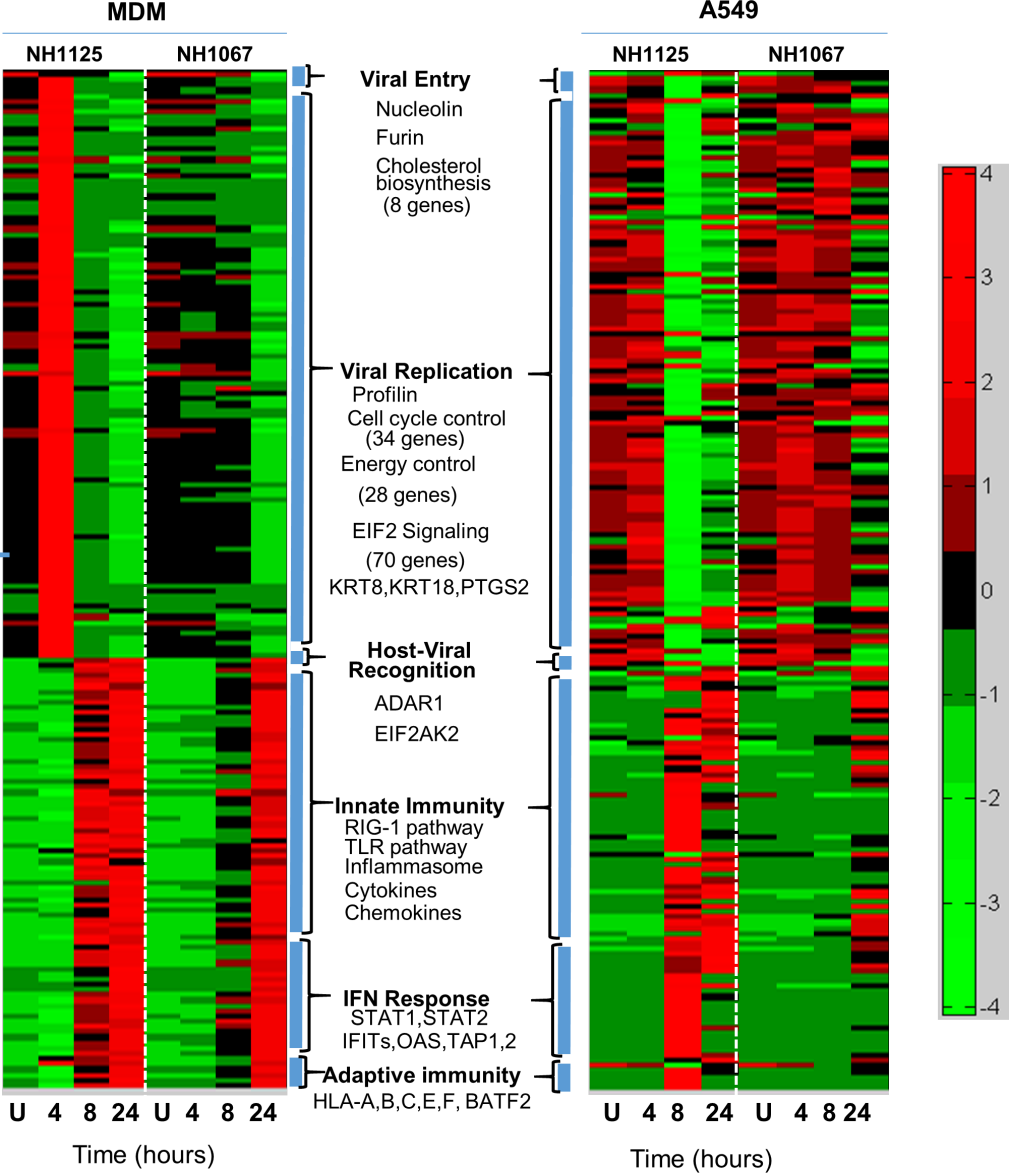
Host gene expression in response to RSV infection. A549 cells or MDM (donor #64) were infected with NH1067B and NH1125B. RNA was extracted at the designated times post infection and gene expression was determined by RNA-SEQ. Individual genes are in rows and variations in expression are depicted using the color scale on the right of the figure. Expression of genes favorable for virus infection (viral entry and viral replication) and expression of genes in response to viral infection (host-viral recognition, innate immunity response, interferon (IFN) response and adaptive immunity) are clustered and relative expression induced by each virus is presented. Some of the genes and biological and biochemical pathways assessed are listed in each category. Mock infected (U) cells were used as controls.

To explore in more detail the differences in induction of cellular gene expression by NH1125B and NH1067B, whole transcriptome analyses were performed at various time points after infection of A549 and MDM (donor #64) cells (Fig 3). Modulation of host gene expression favorable to viral entry and replication were apparent at 4.0 hours post infection in MDM infected with NH1125B though this cellular response was not observed in MDM infected with NH1067B. Genes involved in the response to viral infection, innate immune response, response to interferon and the adaptive immune response were up-regulated in NH1125B-infected MDM by 8.0 hours post-infection whereas this response was, for the most part, delayed in NH1067B-infected MDM. Overall, the patterns of gene expression differed in A549 cells. While there were distinct differences between cells infected with the 2 viruses, particularly at 8.0 hours post infection, there was minimal up regulation of viral recognition, innate immunity, interferon response or adaptive immunity genes in the epithelial cell line, which contrasted sharply with the response of MDM cells infected in an identical fashion.

### Time dynamics of host response to infection

**Fig 4:**
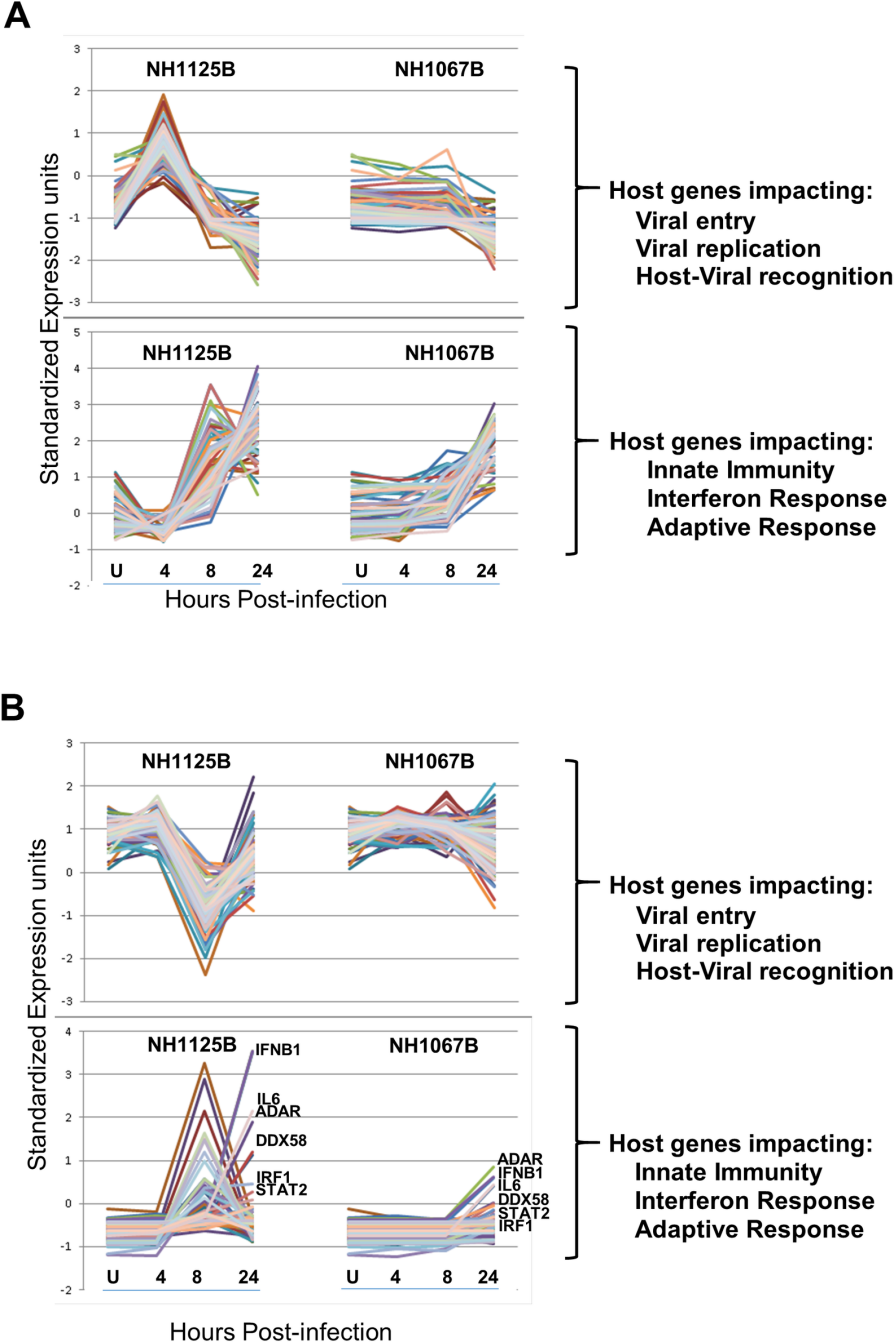
Comparison of time dynamics of cellular genes in response to infection with RSV NH1067B or NH1125B. MDM (donor #64) (A) or A549 cells (B) were infected with NH1067B and NH1125B. RNA was extracted at the designated times post infection and assayed by RNA-SEQ. Time dynamics of expression of genes favorable for virus infection (viral entry and viral replication) and the innate immune response to viral infection (host-viral recognition, innate immunity response, interferon (IFN) response and adaptive immunity) are shown separately in each graph. Representative genes are labeled.

There are distinct patterns of host cell gene expression induced by these 2 clinical isolates at different times post-infection (Fig 4; based on the same data set as Fig 3)). Genes favorable for viral entry and replication were up regulated at 4 hours in NH1125B-infected MDM as compared to NH1067B-infected MDM. These same genes were expressed at high levels in A549 cells prior to infection and down-regulated in NH1125B-infected A549 cells at 8 hours but not by NH1067B. In both A549 and MDM cells infected with NH1067B, there was far less variability over time of host genes impacting innate immunity, interferon response and the adaptive immune response as compared to NH1125B infected cells (Fig 4). Globally, host cell gene activation and suppression occurs earlier during infection with NH1125B as compared to NH1067B (S4 Fig). The number of genes up or down regulated by NH1125B were much greater during early times post infection as compared to NH1067B (see Fig 4, S1 Table, S4 Fig). For example, at 4.0 hours post infection of MDM, 533 genes were up-regulated in NH1125B infected MDM of which only 19 were up-regulated in NH1067B infected MDM (S1 Table, S4 Fig). Likewise, at that time point, 485 genes were down-regulated during NH1125B infection of which none were down-regulated in NH1067B infected cells (S1 Table, S4 Fig). The only gene up regulated in NH1067B-infected MDM that was not upregulated in NH1125B-infected MDM at 4.0 hours post infection was IFIT2. Among the genes upregulated at 24 hours in NH1125B-infected MDM but not NH1067B-infected MDM was *JUN*, a component of the transcription factor AP-1, which is a mediator of pro-inflammatory cytokine production [24]. (For an in depth description of the genes up and down regulated in virus infected MDM, see Panel A-E in S5 Fig).

### Immune activation in response to viral infection is host specific

**Fig 5:**
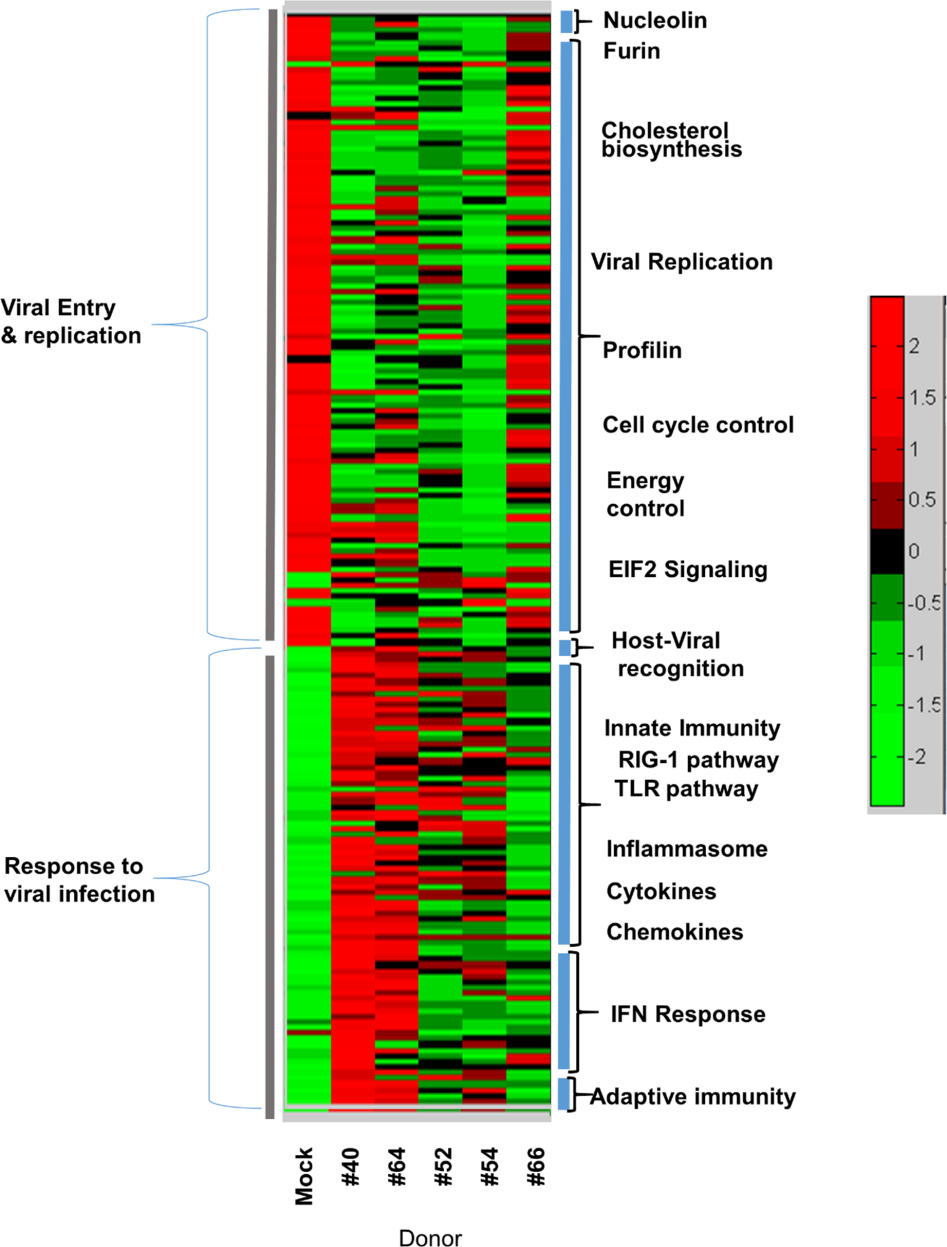
Host-associated differences in response to RSV infection. MDM obtained from 5 donors (#40, #64, #52, #54, #66) were infected with NH1125B. At 24 hours post infection RNA was extracted and gene expression profiles were determined by RNA-SEQ. This heatmap includes expression of genes supporting virus infections (viral entry and replication; see Fig 3) and expression of genes in response to viral infection (host-viral recognition, innate immunity, interferon response and adaptive immunity; see Fig 3).

In order to determine whether the response to virus infection was uniform among individual donors, we infected MDM from several healthy donors and defined the host cell gene activation profiles. Using the same gene clustering approach as described for Fig 3, we identified the transcriptional profiles of MDM in response to NH1125B infection. As shown in Fig 5, there was diversity in the host response of donors in response to RSV infection at 24 hours. While some donors appeared to have a robust innate response (e.g. donor #40), others did not (e.g. donor #66).

### Immune activation to RSV is strain specific

**Fig 6:**
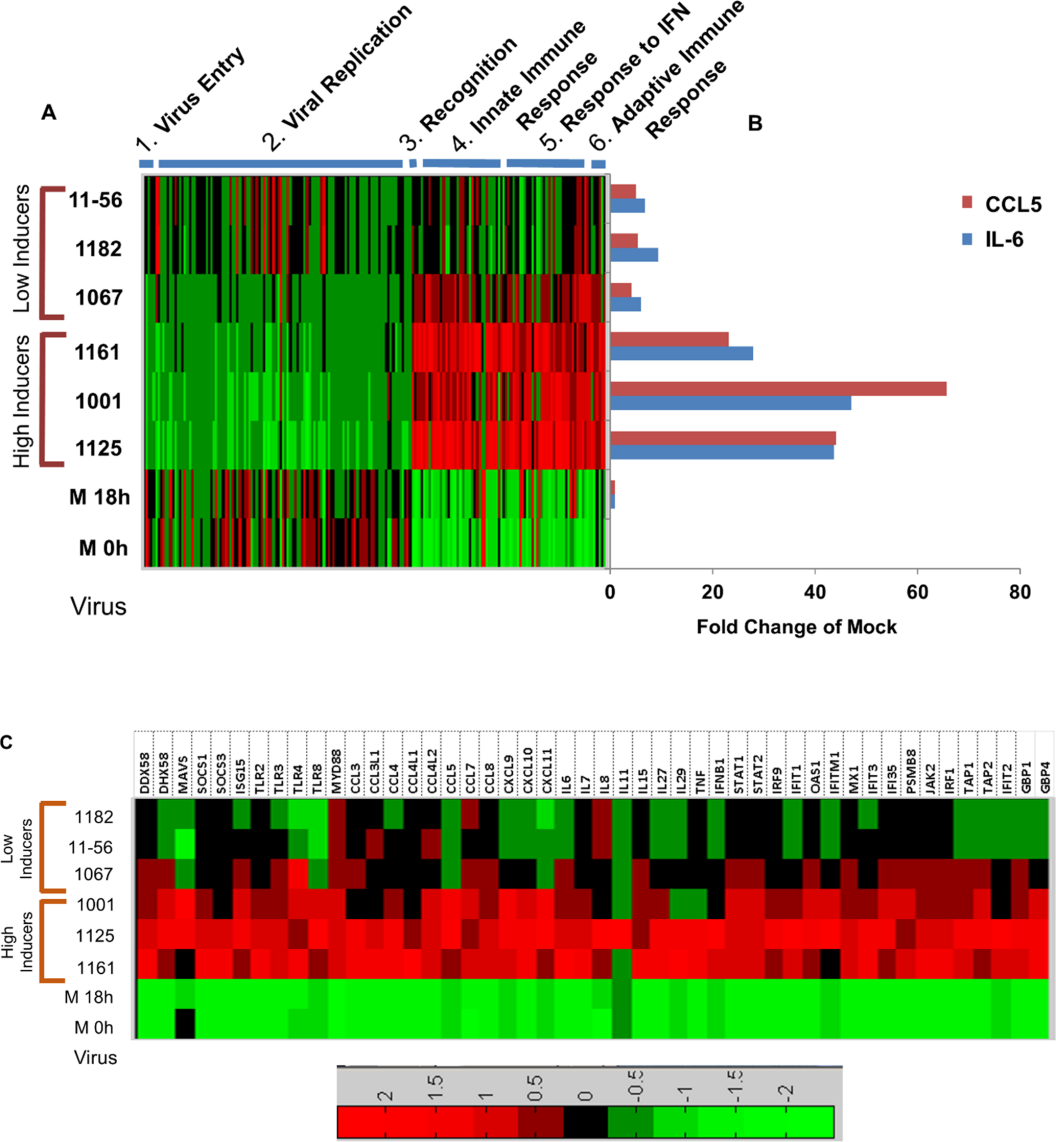
Functional fingerprint of cellular gene expression in MDM infected with multiple clinical isolates of RSV subtype B. (A) Transcriptional heatmaps of cellular genes of MDM (donor #66) infected with RSV. RNA was extracted 18 hours post infection and gene expression was determined by RNA-SEQ. Each row represents a virus (18 hours post infection) or mock infected cells (M) processed at 0 or 18 hours. Clinical isolates include subtype B strains NH1125B (1125), NH1001B (1001), NH1161B (1161), NH1067B (1067), NH1182B (1182), and TX11-56B (11–56). Viruses were designated as “low” or “high” inducers based on their capacity to induce IL-6 and RANTES as shown in the graph (B). IL-6 and RANTES secretion in response to viral infection was measured by BioPlex. (C) Heatmap demonstrating the expression of a subset of cellular genes in MDM infected with clinical isolates of RSV. These genes were selected based on published data indicating that their expression can be regulated by the RSV G protein and or interferon-stimulated gene.

MDM from a single donor (donor #66) were infected with a variety of subgroup B isolates. RNA was extracted at 18 hours post-infection and gene expression was determined by RNA-SEQ. The 18 hour time point for this experiment was chosen because the greatest fold difference in cytokine and chemokine induction between the NH1125B and NH1067B was at this time point (see Fig 1A and 1B) and transcriptome data (S1 Fig) demonstrated differences at this time point. Functional fingerprint of the dynamic changes in the genes described in Fig 3 (above) are shown in the heatmaps displayed in Fig 6. There were marked differences in expression across viral strains. Viral strains which induced relatively high levels of IL-6 and CCL5, (NH1125B, NH1001B and NH1161B) (Fig 6A and 6B designated as “high inducers”), had similar dynamic changes in overall gene expression whereas viral strains that induced relatively lower concentrations of IL-6 and CCL5, (NH1067B, NH1182B, TX11-56B) (designated as “low inducers; Fig 6A and 6B), displayed similar functional fingerprints and relatively little induction of genes that are typically expressed in response to viral infection. In all, it appears that there is concordance between induction of IL-6 and CCL5 (as measured by BioPlex) and the functional fingerprints of cellular gene expression induced by specific RSV viruses.

The expression of several cellular genes is purported to be influence by the RSV G glycoprotein (for comprehensive review see Oshansky, et al [25]), including several TLRs, interferon and *STAT*-related genes. Functional fingerprint analyses of the expression of these genes during viral infection are presented in Fig 6C. Again, the viruses appear to segregate in a similar fashion based on the expression of these genes. Thus, NH1161B, NH1001B and NH1125B, inducing higher levels of expression of these genes as compared with the low inducers subgroup B strains. Among the genes up-regulated by the “high inducers” are several key components of antiviral pathways including *STAT1*, *STAT2*, *MYD88*, *MAVS*, *TLRs* among others.

### Mapping of the induction phenotype in the RSV genome

**Fig 7:**
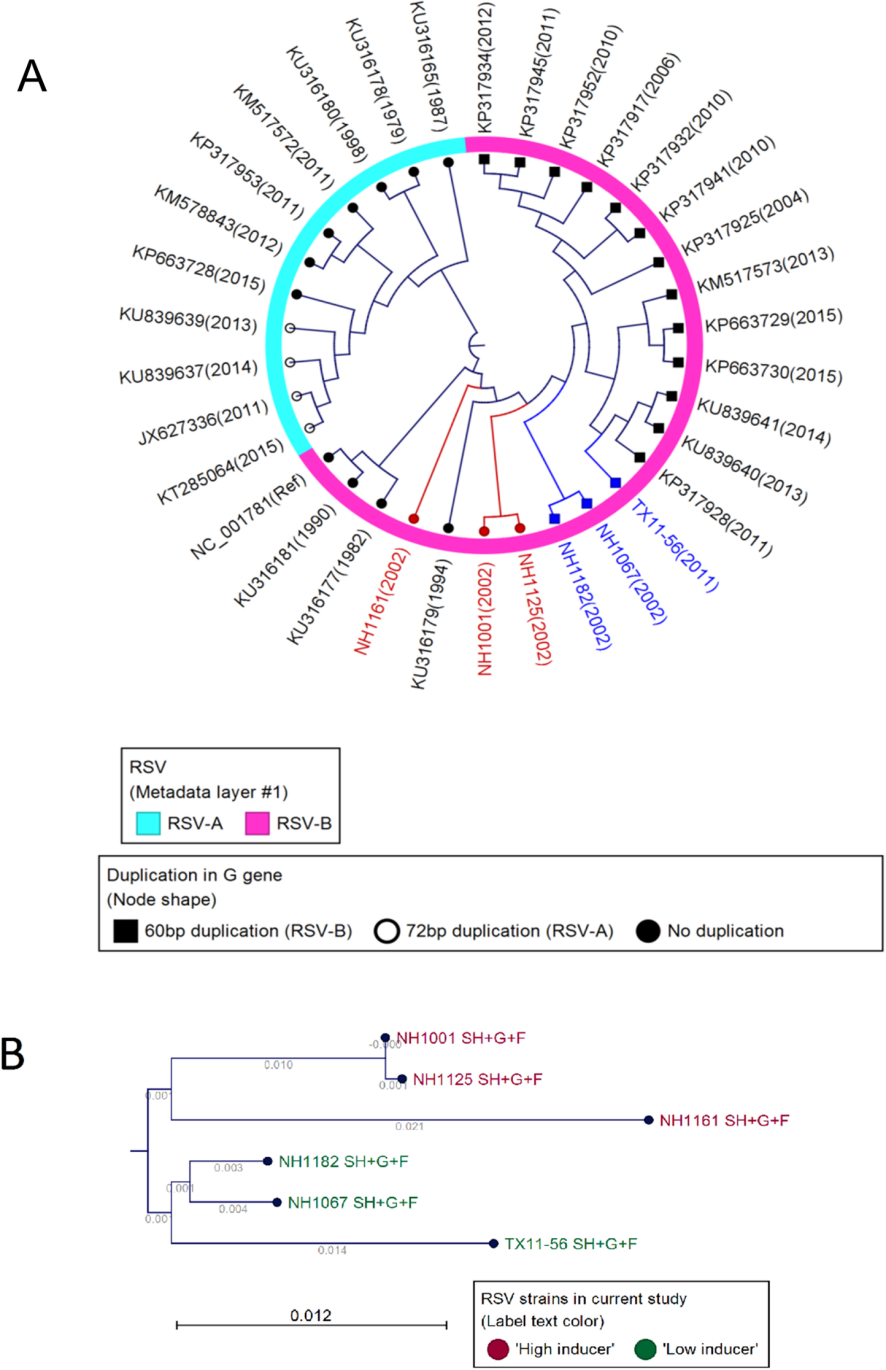
Phylogeny of RSV isolates and correlation to the induction phenotype within the viral genome. (A) Phylogenetic analysis of 33 RSV A and B strains. Analysis was based on the whole genome sequence of each virus. The portion of the dendrogram circle in pink represents B strains and the portion of the dendrogram circle in light blue represents A strains. “High inducers” are in red text and “low inducers” are in blue text. (B) Phylogenic analysis based on the region of the viral genome containing the sequential genes *SH*, *G* and *F*. Sequence and phylogenic analysis suggested that the induction phenotype (“high” vs. “low”; see Fig 6) mapped to this region of the genome. Based on these analyses, segregation of the viral isolates correlated with the cytokine/chemokine induction phenotype.

**Fig 8:**
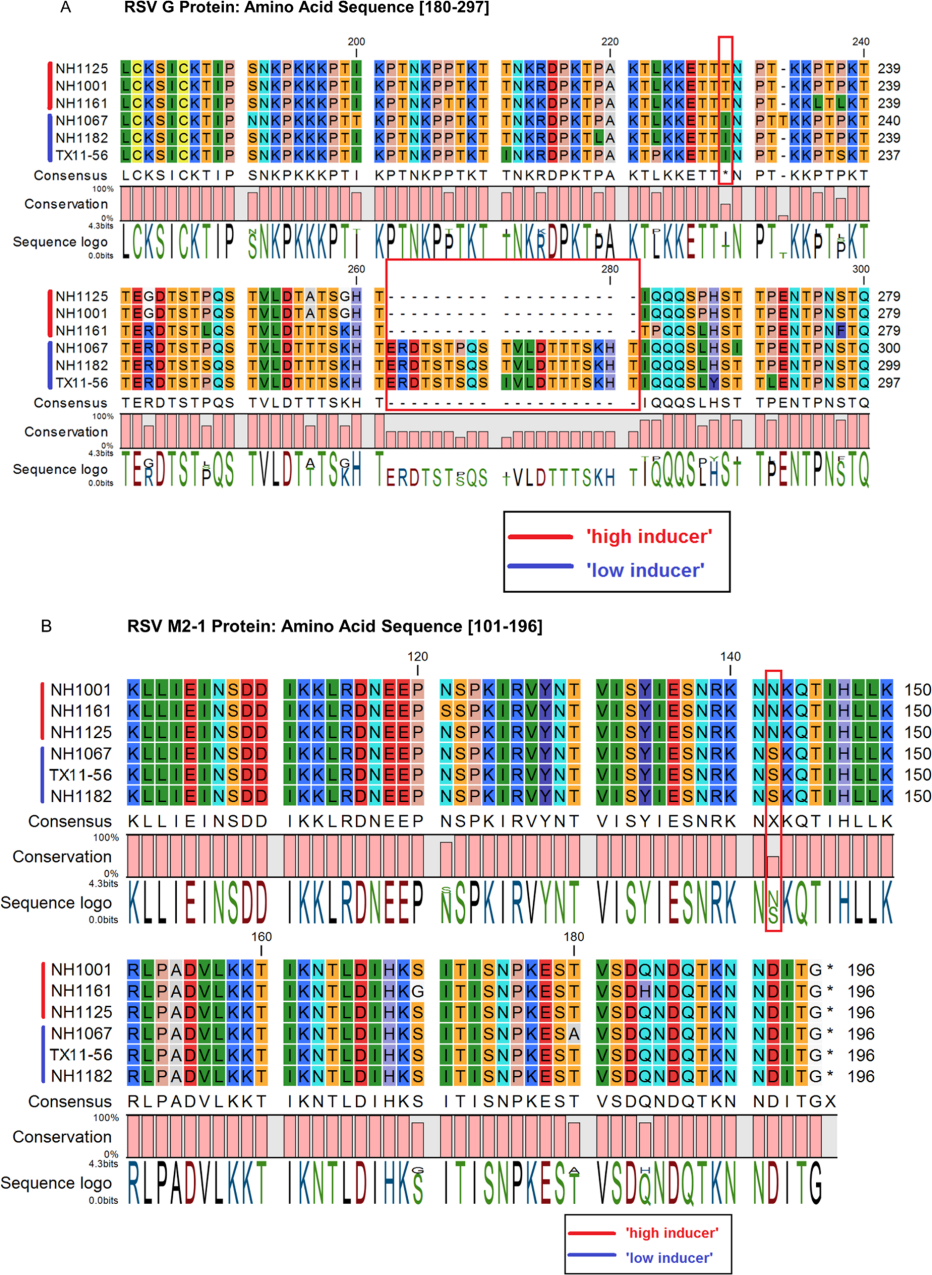
Sequence analyses of RSV isolates and correlation to the induction phenotype within the viral genome. (A) Amino acid sequence of a portion of the G protein of clinical isolates of RSV B. Amino acid sequence, consensus sequence and amino acid number are displayed. The G gene of “low inducers” contains a 20 amino acid duplication (corresponding to the 60 base duplication) absent in “high inducers”. Like the duplication, amino acid residue at position 229 corresponds to viral induction phenotype (isoleucine in “low inducers” and threonine in “high inducers”). (B) Amino acid sequence of a portion of the M2-1 protein of clinical isolates of RSV B. Amino acid sequence, consensus sequence and amino acid number are displayed. Amino acid residue at position 142 corresponds to viral induction phenotype (serine in “low inducers” and asparagine in “high inducers”).

Our data indicate that clinical isolates of RSV are highly variable in their capacity to stimulate or inhibit the innate cellular immune response and that these functional variations are controlled by structural changes in the viral genome. Fig 7A presents a phylogenetic analysis of the whole genome sequences of 33 subgroup A and B strains and demonstrates that the strains examined in this investigation were related to subgroup B strains identified in different years. Detailed phylogenetic analysis of the 6 clinical isolates (Fig 7B) revealed that the induction phenotype (“high” vs. “low”) mapped within the *SH-G-F* gene region (Fig 7B). Sequencing of that region indicated that the phenotype mapped to the RSV G gene (Fig 8A). Sequence analysis of all of the genes in the viral genome revealed a polymorphism in the *M2-1* gene (Fig 8B) that also segregated with induction phenotype. The induction phenotype did not map to any other non-synonymous polymorphisms in any of the other genes in the viral genome. The polymorphisms that correlated with the induction phenotypes in the *RSV G* gene were the 60 base duplication (Fig 8A, boxed) and a single amino acid substitution at position 229 (Fig 8A, boxed) which corresponds to a threonine in “high inducers” and an isoleucine in “low inducers” and a single amino acid substitution at position 142 in the M2-1 protein which corresponds to an asparagine in “high inducers” and a serine in “low inducers” (Fig 8B). The 60 base duplication in the *G* gene of RSV subgroup B isolates was first described in RSV strains identified in South America [26]. Viruses containing this duplication have nearly completely displaced viruses lacking this duplication [27] suggesting an evolutionary advantage for this particular genetic polymorphism. Taken together, these data indicate that viruses containing the duplication and or a threonine at position 229 in the G protein and or the serine at position 142 in the M2-1 protein, such as NH1067B, are more successful in inhibiting the innate immune response early in the viral replication cycle and that this property affords these viral strains with an evolutionary fitness advantage over strains that lack these elements. GenBank accession number for the RSV isolates used in this study are as follows: JQ582844 (NH1067B), JQ582843 (NH1125B), MF185751 (NH1161B), MF185752 (NH1182B), MF185753 (TX11-56B) and MF185754 (NH1001B).

### RSV gene expression during infection of A549 cells and MDM

Several studies of RSV and related paramyxoviruses have demonstrated that there is a gradient of transcription across the viral genome from the genomic 3’ end to the genomic 5’ end such that, for RSV, the level of transcription of the genes decreases in the following gene order: *NS1-NS2-N-P-M-SH-G-F-M2-1/M2-2-L* [28, 29]. RSV gene expression in infected MDM cells was determined using RNA-SEQ reads that did not map to the human genome. At 18 hours post-infection (donor #66), for both NH1067B and NH1125B, there appeared to be relatively active gene expression for all genes except for the polymerase gene (L) (S6 Fig). Consistent with the use of polyadenylated transcripts for this type of analyses, there were regions of the viral genome with low transcriptional activity, specifically intergenic region, in particular, at the *NS1*/*NS2* and *SH*/*G* junctions for both viruses. To further explore the dynamics of RSV gene expression over time, RNA was extracted from infected cells, both A549 and MDM (donor #64), at numerous time points post-infection gene expression was determined using RNA-SEQ. In all, there appeared to be an overall gradient of gene expression (S7 Fig). However, the expression of the *P* gene or the *G* gene appeared to be highest among all genes at nearly every time point for both viruses. There was a marked reduction in viral gene expression in NH1125B-infected MDM at 4.0 hours post-infection as compared to NH1067B infected MDM and this coincided with the most dramatic difference in host gene expression as shown in Fig 3. This difference was not observed in infected A549 cells (S7 Fig). Consistent with the data presented in S6 Fig, expression of the *L* gene was substantially lower than other viral genes at each time points.

## Discussion

Here, we describe an investigation of clinical isolates of RSV in cells of human origin, including MDM from a variety of donors. We chose human MDM because macrophages represent a key cell lineage in the control of RSV infection [30] and are responsible for much of the inflammatory response during infection with RSV [17]. Furthermore, there is mounting data indicating that the innate immune response to RSV drives pathogenesis [31] and that immune dysregulation in response to RSV, as defined by gene expression profiles of children infected with RSV, contribute to pathogenesis [32]. Our results demonstrate that the patterns of host cell gene expression differ in cells infected with clinical strains of RSV, strongly suggesting that viral genomic polymorphism underlie, in part, the differences in the severity of clinical disease observed in infants and young children. These data are consistent with previous studies that demonstrate that A549 cells infected with viruses collected from children with severe disease produced higher levels of IL6 and CCL5 (RANTES) than cells infected with viruses obtained from children with mild disease[33]. While some of the viruses (e.g. NH1125B) induced a strong inflammatory response, consistent with gene profiles observed in animal models [34, 35], several viruses (e.g. NH1067B) did so to lesser extent suggesting that these viruses may possess different disease potential. It appears that NH1067B and other NH1067B-like viruses are more efficient at inhibiting innate immune responses, including the induction of cytokines early in viral infection and that this may contribute to the viral pathogenicity. Indeed, blood transcriptome profiles of RSV-infected children has been used to predict prognosis of severe disease supporting the use of these types of genomic and transcription signatures studying viral pathogenesis[36], and in the case of this study identifying mapping elements in the viral genome that induce or perhaps suppress the innate immune response.

The study of clinical isolates and naturally-occurring variants has been an essential element in defining pathogenesis or the role of specific viral genes in human infections. For example, the finding of an HIV strain that contained a spontaneous deletion in the *nef* gene in a long term non-progressor and a cohort of individuals who received blood transfusions from this individual was the essential observation in defining the role of this gene in disease[37]. Up until the identification of this *nef*-deleted mutant, the *nef* gene was thought to be an accessory gene (i.e. not required for virus replication *in vitro*) with an unclear role(s) in pathogenesis [38]. It is now well defined that the *nef* gene down regulates several cell surface molecules and that this down-regulation is essential for pathogenesis and virulence [39]. Ebola virus Reston, closely related to the highly virulent Ebola virus Zaire, appears to be non-pathogenic in humans. By comparing the sequences of the pathogenic and non-pathogenic viruses, potential Ebola virulence factors, such as the virion glycoprotein, were identified [40]. Therefore, it is a reasonable hypothesis that clinical strains of RSV differ in their pathogenic potential and that the variability of clinical disease observed in RSV-infected children may be due, in part, the variability in virulence factors encoded by the virus. In fact, among laboratory strains of RSV, the capacity to inhibit TLR-dependent and TLR-independent responses in human plasmacytoid dendritic cells differs [41] further supporting our hypothesis.

**Fig 9:**
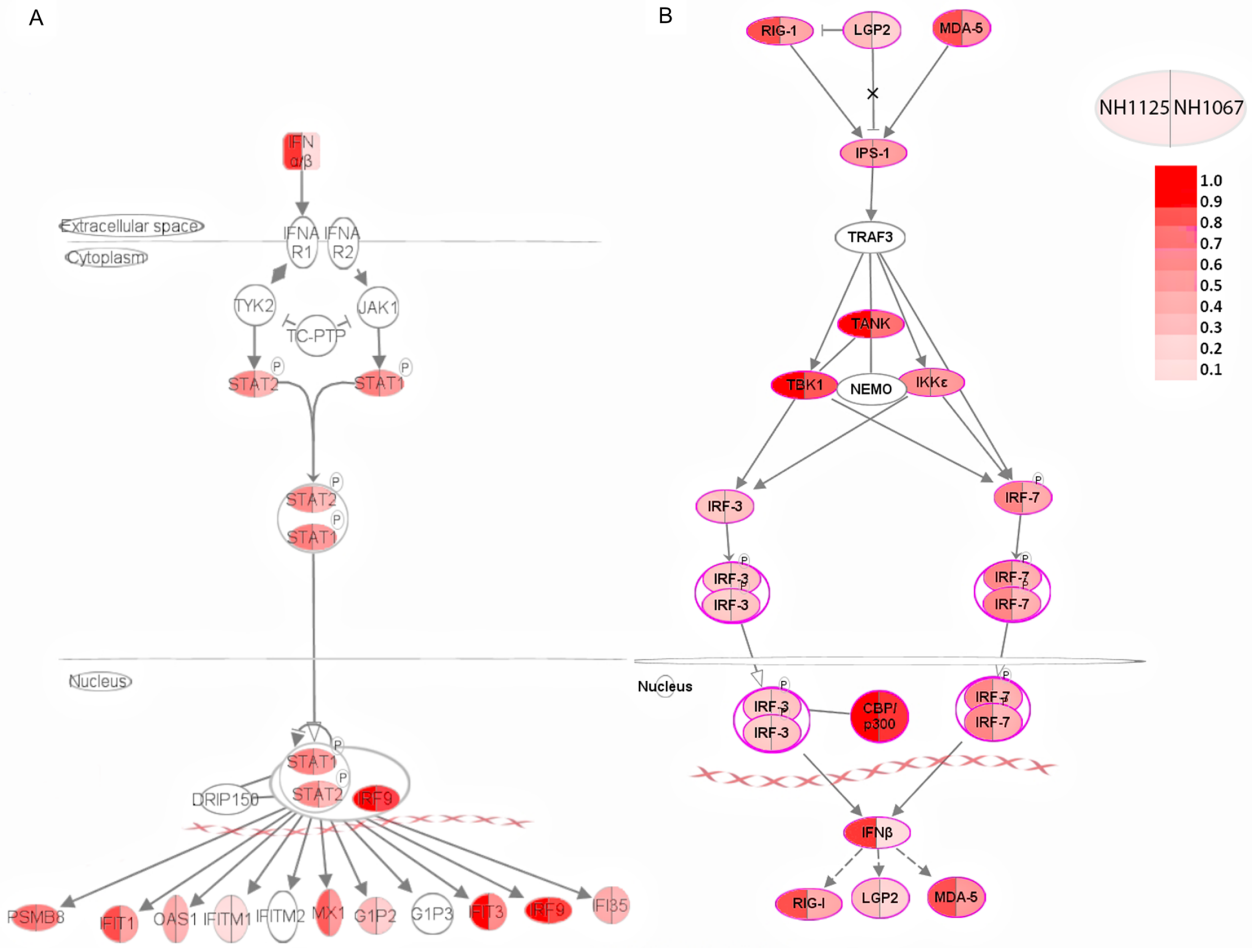
Pathway analysis of interferon stimulated genes after RSV infection. Interferon stimulated genes are organized based on known established pathways and subcellular locations. Symbols are bi-colored with the left half characterizing genes activated by NH1125B and the right half genes activated by NH1067B. The differences in color intensity reflect differences in the expression levels of the genes by each virus. Data for this figure were obtained at 8 hours (A) and 24 hours (B) post infection of MDM cells.

The activation of the interferon pathways by NH1125B and NH1067 differ quantitatively (Fig 9) with, based on our transcriptome data, predictable downstream gene activation sequelae. In vitro studies using recombinant virus have demonstrated that the non-structural genes of RSV, *NS1* and *NS2*, possess anti-interferon activities and are, at least to some extent, part of the RSV encoded anti-viral armamentarium [42–45]. However, the differences in cytokine induction observed between clinical strains NH1125B and NH1067B are unlikely to be associated with polymorphisms in the *NS1* gene of these viruses. Overall, there is a single silent nucleotide polymorphism present in the *NS1* gene between NH1125B and NH1067B. There are 4 nucleotide polymorphisms in the *NS2* gene between NH1125B and NH1067B, 3 of which are silent and 1 which results in a methionine (NH1125B) to leucine (NH1067B) at position 10 ([14], GenBank JQ582843, JQ582844). These *NS1* and *NS2* variants did not correlate with the magnitude of innate immunity they induced suggesting that there may be other components of the viral genome which contribute to interferon suppression or induction.

The RSV *G* gene has been implicated in directing the host innate immune response to the virus [46–56]. The genetic diversity in RSV strains is most pronounced in the *G* gene [57] and, consistent with that observation, the viruses examined in this study displayed the greatest level of diversity in this gene. Notably, NH1067B and the other low-inducing viruses (NH1182B and TX11-56B) all contain a 60 base duplication (not present in NH1125B) which was initially described in isolates in South America [26]. The duplication in the *G* gene has been reported to augment viral attachment and fitness [58] perhaps providing these viruses with an evolutionary advantage over viruses that do have this duplication. Indeed, subgroup B viruses harboring this duplication have nearly displaced subgroup B viruses that lack this duplication [27]. Our data correlate the presence of this duplication with “low induction” phenotype however, it is not clear whether this duplication and/or the single amino acid polymorphism at position 229 of the G protein or the amino acid substitution at position 142 of the M2-1 protein are solely responsible for the “low inducer” phenotype. The CX3C motif (amino acid 182–186) was identical in all viruses [59]. The M2-1 protein acts as a transcriptional elongation factor that prevents premature termination during transcription and functions as an anti-termination factor as the polymerase switches from the transciptase activity to a genomic replicase activity [60]. Based on the RNA-SEQ data (S6 and S7 Figs), there does not appear to be a global difference in the gene transcript profile of NH1125B as compared to NH1067B suggesting that this amino acid polymorphism may have an alternative role (if any) in the viral phenotypes. Nonetheless, the contribution of these amino acid polymorphisms will need to be deciphered using alternative approaches. Furthermore, non-synonymous or non-coding region polymorphisms may play a role in the observed differences in the viral phenotypes.

Transcriptome analyses are a powerful tool to define the cellular and organismal response to pathogens. Likewise, this powerful technology can be used to define the kinetics of viral gene expression during infection. Here we showed that the expression of viral genes was consistent with previous studies using hybridization technologies. For example, our data revealed that there was relatively low expression of the *L* gene. This is not entirely unexpected as this gene encodes the enzymatic RNA-dependent RNA polymerase, which is contained within virions and delivered into the cytoplasm upon infection, and is likely needed in relatively small quantities in infected cells to perform its transcriptional and replicative functions. Our data are consistent with previous studies that have demonstrated that there is a gradient of transcription of viral genes across the viral genome from the 3’ end (of the negative strand beginning with a leader sequence and the *NS1* gene) to the 5’ end [28, 29]. This pattern is present in both infected A549 cells and MDM (S6 and S7 Figs). However, there are 2 genes that appear to stand out from this pattern, the *G* gene as noted above, and the *P* gene. In both A549 cells and MDM, RSV G expression was the highest among all viral genes in NH1125B infected cells at late times post infection (8.0 and 24 hours, S7 Fig). This was not the case with NH1067B. The significance of *G* gene expression and its relationship to host cell gene expression remains to be determined.

Many human genes have been implicated in the host innate immune response to viral infection. RIG-I (encoded by the DDX58 gene), a pattern recognition receptor for viral RNA, and MAVS, which mediates the activation of NFKB and IRF3 in response to viral infection [61], both key molecules in the cellular innate immune response, are activated to a lesser extent by NH1067B and other “low inducers” as compared to NH1125B and other “high inducers” at early times post infection (Fig 6C). These data imply that “low inducer” viruses such as NH1067B either suppress or evade early recognition of the innate immune system, perhaps resulting in an evolutionary advantage for these viruses that may have contributed to the increased frequency of RSV clinical isolates with this G gene duplication over the past decade. Clinical studies have identified polymorphisms in specific innate immune response genes that appear to be associated with increased susceptibility to severe RSV disease. For example, the 7 genes listed in Table 1, which are differentially induced by NH1125B and NH1067B, have been implicated in clinical investigations to be linked to severity of illness further supporting the hypothesis that feral strains of RSV differ in disease potential. Genes linked to host defenses are also differentially expressed in response to infection with clinical isolates of RSV. For example, the expression of *JUN*, which encodes a component of the transcription factor AP-1, which is a mediator of proinflammatory cytokine production [23], is stimulated in the early stages of infection of MDM with NH1125B but not NH1067B. Single nucleotide polymorphisms in *JUN* has been associated with susceptibility of children to RSV bronchiolitis [62]. Large data sets, incorporating in vitro and clinical data, have identified a small number of genes upregulated during RSV infection (Table 2). The expression of these genes differs in cells infected with NH1125B as compared to NH1067B. While the function of some of these genes in RSV disease remains unknown, it is likely that the upregulation of expression is a key component of the cellular innate immune response. Two distinct patterns of induction of >200 genes by RSV were observed in cells obtained from 5 human donors (Fig 5) indicating that variations in the human genome impact the magnitude of the innate immune response induced by RSV. Taken together, the data presented suggests that the viral-host couple may determine the severity of disease with both viral and host genetic factors involved in the complex dynamics of pathogenesis.

**Table 1:** Genes associated with RSV pathogenesis and expressed differently in response to NH1125B or NH1067B infection.

#	Gene name	Figure	Protein name	Protein Function	Claims for Severity / pathogenesis (reference #)
**1**	MX1	Fig 6C	Myxovirus Resistance Protein 1	This gene encodes a guanosine triphosphate (GTP)-metabolizing protein that participates in the cellular antiviral response. The encoded protein is induced by type I and type II interferons and antagonizes the replication process of several different RNA and DNA viruses	[63, 64]
**2**	TLR8	Fig 6C	Toll Like Receptor 8	Toll-like receptor (TLR) family plays a fundamental role in pathogen recognition and activation of innate immunity and mediates the production of cytokines necessary for effective immunity. This gene is predominantly expressed in lung and peripheral blood leukocyte	[62, 65]
**3**	CCL7/ MCP-3	Figs 6C and 2A	Chemokine (C-C Motif) Ligand 7	Chemotactic factor that attracts monocytes and eosinophils, but not neutrophils	[66]
**4**	CCL8/ MCP2	Figs 6C+2A	Chemokine (C-C Motif) Ligand 8	Chemotactic factor that attracts monocytes, lymphocytes, basophils and eosinophils. May play a role in neoplasia and inflammatory host responses. This protein can bind heparin.	[65, 67]
**5**	STAT1	Fig 6C	Signal Transducer And Activator Of Transcription 1	Signal transducer and transcription activator that mediates cellular responses to interferons (IFNs), cytokine KITLG/SCF and other cytokines and other growth factors.	[64, 65, 67]
**6**	IL7	Fig 6C	Interleukin 7	Hematopoietic growth factor capable of stimulating the proliferation of lymphoid progenitors. It is important for proliferation during certain stages of B-cell maturation.	[23]
**7**	IRF7	S4 Fig	Interferon Regulatory Factor 7	Key transcriptional regulator of type I interferon (IFN)-dependent immune responses and plays a critical role in the innate immune response against DNA and RNA viruses. Regulates the transcription of type I IFN genes (IFN-alpha and IFN-beta) and IFN-stimulated genes (ISG) by binding to an interferon-stimulated response element (ISRE) in their promoters	[64]

**Table 2:** List of genes up-regulated in high inducers infection vs. low inducers and were identified in Reference 64 as up-regulated in blood cells in RSV infected infants (relative to control).

#	Gene name	Figure
**1**	DHX58	Fig 6C
**2**	IFIT1	Fig 6C
**3**	OAS1	Fig 6C
**4**	IFIT3	Fig 6C
**5**	IFI35	Fig 6C
**6**	ISG15	S4 Fig
**7**	UPS18	S4 Fig

In conclusion, we have demonstrated that clinical isolates of RSV are heterogeneous in regards to biological properties, including the induction or suppression of host cell innate immune responses. Overall, it seems that the timing of the induction of the innate immune system is dependent on the virus while the magnitude of the response is dependent on the host. Early activation of the innate immune system, as is the case with NH1125B, likely has implications for disease severity and pathogenesis. Experimental infection of non-human primates with highly pathogenic avian influenza H5N1 demonstrated that disease severity is associated with differential gene expression early in infection. Overall, the induction of interferon-induced upregulation of genes related to innate immunity, apoptosis and antigen presentation during early stages of infection was limited in severe infection[68]. Whether this is the case with RSV strains identified here remains to be determined. Our data indicate that viral sequences may dictate pathogenesis and, in fact, may explain, in part, the varying degrees of severity of illness observed in infants and young children. Furthermore, we show that the investigation of clinical isolates, which possesses naturally-occurring polymorphisms, may lead to the identification of sequences that underlie viral virulence. Whether a more efficient suppression of innate immunity correlates with increases or decreases in viral pathogenesis remains to be determined. Our ongoing investigation of the variations in the RSV genome may lead to the development of novel antiviral agents and approaches.

## Materials and methods

### Virus and cells

RSV clinical isolates were obtained from RSV-infected individuals, as described previously, in New Haven, CT [69] and Dallas, TX [14]. Isolates were plaque-purified, concentrated, quantified by plaque titration and working stocks prepared as described previously [14] and elsewhere [70]. Viral stocks were prepared with a low inoculum (m.o.i. of 0.01–0.05) to minimize the production of defective interfering (DI) particles. Further, the number of passages of virus in cell culture was limited to prevent the potential viral adaption to cell culture. For all experiments, a multiplicity of infection (m.o.i.) of 0.2–0.33 was used. The m.o.i. was dependent upon the titers of the stocks of each of the clinical isolates. For each individual experiment, the m.o.i of each virus was identical. All specimens from which viruses were obtained were submitted as part of routine care. Only left over material was used for viral propagation. Collection of specimens from the Clinical Virology Laboratory at Yale-New Haven Hospital was approved by the Yale University Human Investigations Committee. The single isolate from Dallas, Texas was propagated from a de-identified clinical specimen obtained from the Clinical Microbiology Laboratory at Children’s Medical Center, Dallas. Collection and use of clinical isolates followed all institutional requirements and guidelines and was consistent with policies and regulations for the use of patient derived materials.

A549 (CCL-185) cells were obtained from the American Type Culture Collection (Manassas, VA) and cultivated in F-12 Kaighn’s modification media with 10% fetal bovine serum. Primary monocyte-derived human macrophages (MDM) were prepared as follows: peripheral blood mononuclear cells (PBMC) from healthy human (white Caucasian female) donors were enriched by density gradient centrifugation through a Ficoll- Hypaque gradient. For generation of MDM, PBMCs were plated in tissue culture treated dishes and incubated for 2 hours at 37°C in a humidified CO_2_ incubator. To obtain monocytes, non-adherent cells were discarded by washing 3 times. RPMI-1640 with 10% FBS, 2mM L-glutamine, 10 mM HEPES, 1 mM sodium pyruvate, 100 U/ml penicillin, 100 μg/mL streptomycin and 50ng/ml M-CSF were added to the dish. The culture media which contained fresh M-CSF were replaced every 2 days. MDMs were harvested on day 7.

### Ethics statement

Human peripheral blood mononuclear cells (PBMCs) were obtained from adult healthy donors in accordance with the guidelines established by the Institutional Review Board (IRB) of the University of Texas Southwestern Medical Center (UTSW). All subjects gave their written informed consent and research protocols and methods employed were approved by the UTSW IRB.

### Cytokine assays

Monolayers of A549 cells or MDM were infected with sucrose-purified clinical isolates of RSV. After 90 minutes of infection, the inoculum was removed, the cells were washed with serum-free media and fresh media (F-12 Kaighn’s modification media for A549 cells; RPMI1640 for macrophages) containing 5% FBS was added to the infected monolayers. M-CSF (50 ng/ml) was added to the media of the macrophages cultures. For cytokine analysis, supernatants were collected, clarified by centrifugation and snap frozen in liquid nitrogen and stored at -80°C until the specific assay was performed. Concentrations of IL-6 and CCL5 were measured using Bio-Plex Pro™ with conjugated magnetic beads according to the manufacturer’s instructions. Cytokine data were analyzed using Bio-Plex Manager™ version 4.1.1 software.

### Virus neutralization

For neutralization experiments, clinical isolates of RSV were incubated with an RSV-neutralizing monoclonal antibody (Synagis®, MedImmune, Gaithersburg, MD, catalogue # NDC60574) at a final dilution of 1:400 at 37°C for 1 hour prior to infection of MDM. After 90 minutes of infection, the inoculum was removed, cells were washed with serum-free media and incubated for 18 hours with RPMI1640 containing 5% FBS and M-CSF (50 ng/ml). Neutralization of RSV was confirmed by plaque assay. Overall, pretreatment of RSV with Synagis reduced the titer of virus greater than 4 orders of magnitude.

### RNA extraction, RNA-SEQ and gene expression data analyses

Total cellular RNA was extracted from A549 cell or MDM using the QIAshredder columns and RNeasy Mini kit (QIAGEN, Valencia, CA) according to the manufacturer’s recommendations. RNA concentration and quality were determined by Aligent 2100 Bioanalyzer. RNA-SEQ was performed as previous described [71]. Briefly, 0.1 to 1.0 micrograms of high quality purified RNA (RNA integrity number [RIN]>8.5; the RIN is a verified methodology for assessing the quality of RNA for gene expression measurements [72]) were used for producing RNA-SEQ cDNA libraries using TruSeq RNA Library Preparation Kit v2 (Illumina, Inc.) as per the manufacturer’s recommendation. For any given experiment, the amount of RNA used was equal. (Note: replicates using RNA concentrations in the ranges specified did not result in significant differences in transcriptome data.) This included using standard protocols for cDNA synthesis, fragmentation, addition of adaptors, size selection, amplification and QC (Illumina). SE50 single-end sequencing was done using HI-SEQ 2500 (Illumina) with > 18,000,000 reads/sample. Basic data analysis was done using CLC-Biosystems Genomic Workbench analysis programs to generate quantitative data for all genes, including reads per kilobase per million (RPKM) values, unique and total gene reads, annotated transcripts and detected transcripts, median coverage, chromosomal location, and putative exons. The data for the heatmap are expressed in standardized units: Dst = (D—Avr)/STD, where D is level of expression of given gene (RPKM) in given time point, Avr and STD are averaged expression and standard deviation correspondingly for given gene over all conditions presented in the figure.

Methods for data normalization and analysis are based on the use of “internal standards” [73] that characterize some aspects of the system’s behavior, such as technical variability, as presented elsewhere [74, 75]. Internal standard in this context is considered as a large family of genes sharing some useful features for analysis, which in turn are neither dependent on the particular gene sequence nor on the level of expression. The internal standard methodology serves us as a stepping stone to normalization procedure and differential gene expression analysis in a statistically robust manner, to finding functional associations through clustering and networking genes having similar dynamical behavior. The internal standard used for the data normalization and for selection of differentially expressed genes consists of the majority of equally expressed genes obtained with iterative procedure described elsewhere[75]. At the beginning, all genes are represented by their residuals (relatively averaged profile), which after normalization and log transformation lose their sample-dependent individuality as well as their expression level-dependent individuality. Differentially expressed genes were selected as outliers from this standard (normally distributed gene expression residuals) beyond of some statistical thresholds, that was used in our case equal to 1/(approximate number of genes expressed distinctively from background noise) ~ 10e4.

In the analysis of the time dynamics of gene expressions in the infected MDM we first selected all genes demonstrating significantly increased variability (hyper variable expressed genes [HVE]). One of the most important criteria in the selection of HVE-genes and the analysis of their behavior is the choice of the ‘Reference Group’—which is composed of genes expressed above the background of control samples with a low variability of expression (as determined by an F-test). Procedure for establishing the ‘Reference Group’ was described in detail elsewhere [76]. The comparison of these methods with some other normalization and analysis procedures was presented elsewhere[77]. Created initially for the analysis of microarray data they were slightly modified to the needs of RNA-SEQ data analysis.

The two-step normalization procedure and the associative analysis functions are implemented in MatLab (Mathworks, MA) and available from authors upon request. These algorithms are also obtainable from an R package diffGeneAnalysis, available as a part of Bioconductor packages (http://www.bioconductor.org/packages/2.5/bioc/html/diffGeneAnalysis.html).

The study of the time dynamics in gene expressions were used genes whose expression level varied significantly when compared to the variability to that of the ‘reference group’ (denoted hyper-variable (HVE) genes). Details of the statistical selection of these genes and clustering procedures used for analysis of their collective behavior are presented elsewhere [76]. Heatmaps were generated with the Matlab and Spotfire Decision Site 9 (TIBCO, Palo Alto, CA) with gene subsets created from the list of significant genes. Functional analysis of identified genes was performed with Ingenuity Pathway Analysis (IPA; Ingenuity® Systems, Redwood City, CA, http://www.ingenuity.com).

### Quantitative real time PCR (RT-qPCR)

Briefly, 500 ng of RNA was reverse transcribed using the SuperScript ® III First-Strand Synthesis System (Invitrogen) according to manufacturer’s protocol. RT-qPCR was performed using PowerUp™ SYBR® Green Master Mix (applied biosystems) on the QuantStudio 7 Flex Real-Time PCR System (applied biosystems) according to the manufacturer’s directions. Primers were as follows: GAPDH, 5′-TCTCTGCCCCCTCTGCTG-3′ (forward) and 5′-AGTCCTTCCACGATACCAAA-3′ (reverse); IL6, 5′-CACACAGACAGCCACTCACC-3′ (forward) and 5′-CCTCAAACTCCAAAAGACCA-3′ (reverse); CCL5, 5′-CGCTGTCATCCTCATTGCTA-3′ (forward) and 5′-ACACACTTGGCGGTTCTTTC-3′ (reverse); and TNFα, 5′-CTCCTCACCCACACCATCA-3′ (forward) and 5′-GGAAGACCCCTCCCAGATAG-3′ (reverse). GAPDH was used as an internal control. The comparative CT (ΔΔCT) method was used for data analysis. Error bars represent means ±SD.

### Viral phylogenic and transcriptome analyses

Phylogenic analyses were performed and phylogenic trees were generated using CLC-Biosystems Genomic Workbench (Qiagen). In-study RSV strain genomes were assembled by mapping gene reads (described above) to either NH1125B or NH1067B genome that did not match to the human genome [14]. Genome assemblies of representative RSV-A and RSV-B strains used in phylogenic analysis were downloaded from NCBI database. For phylogenic analysis of RSV *SH*-*G*-*F* region, sequences of *SH*-*G*-*F* were selected from assembled genomes described above and used for analysis. Amino acid translation and alignment was also performed using CLC-Biosystems Genomic Workbench (Qiagen).

Gene reads (described above) that did not map to the human genome were used for viral transcriptome analyses. RSV gene expression was normalized for reads per base pair gene length (RPB) as follows: RPB = R/G where R is the total reads and G is the gene length (in bases). The RPB for each RSV gene was normalized to housekeeping gene expression in the corresponding human transcriptome to rule out the possibility of reduced cell number and different read depth. The normalized reads per gene base pair RPKN) is calculated as RPK/N, where N is the normalization of housekeeping genes [78]. List of housekeeping genes includes *TUBB*, *YWHAZ*, *B2M*, *TBP*, *RPLPO*, *RPLP1*, *HPRT1*, *SDHA* and *ACTB*.

## Supporting information

S1 Fig Host gene expression in response to RSV infection. A549 cells or MDM (donor #67 and #68) were infected with NH1067B and NH1125B. RNA was extracted at the designated times post infection and gene expression was determined by RNA-SEQ. Individual genes are in rows and variations in expression are depicted using the color scale on the left of the figure. In MDM heatmap, for each time point, the left column represents donor #67 and the right column represents donor #68. For A549 cells, each column at each time point represents a biological replicate. Mock infected (U) cells were used as controls.
(PPTX)

S2 Fig Pathway induced during RSV infection. Pathway induced during RSV infection (NH1125B) include A) RIG-I; B) TLR; C) Inflammasome; D) IL-6 signaling; E) NF-ĸB; F) Interferon; G) Death receptor signaling; H) DNA damage response and; I) Cell cycle control of chromosomal replication. Red indicates increased transcription and green indicates decreased transcription (based on RNA-SEQ data).
(PPTX)

S3 Fig Induction of IL-6 and CCL5 (RANTES) by clinical isolates of RSV is abrogated by pretreatment of virus with an RSV neutralizing antibody. MDM (donor #96 and #98) were infected with clinical isolate NH1125B (1125) or 1125 pre-treated with an RSV-neutralizing antibody (1125+SYN). As controls, cells were incubated with an RSV-neutralizing antibody (SYN), TLR7 stimulator (R848) or incubated with R848 and SYN (R848+SYN). At 20 hours post-infection, cell culture supernatants were collected and concentration of IL-6 (A, C) and RANTES (B, D) were measured by BioPlex assay.
(PPTX)

S4 Fig Host gene expression following RSV infection. MDM (donor #64) or A549 cells were infected with clinical isolate NH1125B (1125) or NH1067B (1067). At several time points post infection, RNA was isolated and gene expression was determined by RNA-SEQ. In the scatter plot diagrams of cellular gene expression in MDM or A549 cells, each circle represents a specific gene (expressed as reads per kilobase per million (RPKM)) relative to expression of that gene in uninfected cells. Gene expression in cells infected with NH1125B (1125) are displayed as red dots and gene expression in cells infected with NH1067B (1067) are displayed as light blue dots. Genes associated with specific pathways are labeled. Venn diagram displays differential cellular gene expression of MDMs infected with clinical isolates NH1125B (pink), NH1067B (blue) or both (purple) at different time point post-infection compared to mock control. The numbers in the diagram, also displayed in the S1 Table, represent up-regulated genes (numerators) and down-regulated genes (denominators).
(PPTX)

S5 Fig **(A-E). Functional fingerprint of cellular gene expression during RSV infection.** MDM (donor #64) were infected with clinical isolates NH1125B or NH1067B and at several times post-infection, RNA was isolated and cellular gene expression was determined by RNA-SEQ. Genes are clustered based on their functionality which is described on the left side of the figure. The gene expression profiles correspond to the data represented in Figs 3 and 4 and S4 Fig.
(PPTX)

S6 Fig RSV gene expression during infection of MDM. MDM (donor #66) were infected with NH1125B (A) or NH1067B (B) and at 18 hours post infection, RNA was isolated and gene expression was determined by RNA-SEQ. Here, reads that did not map to the human genome were aligned to the viral genome. The Y-axis represents the magnitude of transcription for each region of the viral genome. A map of the viral genome (C) including the coding regions for the RSV genes is displayed beneath the graph of the gene expression. (D) Comparison of the genome sequence of clinical isolates NH1125B and NH1067B. Each vertical bar represents a nucleotide polymorphism between the 2 strains. The "*" represent 1 or 3 base insertions and the triangle designates the location of the 60 base duplication in the G gene of NH1067B.
(PPTX)

S7 Fig Dynamics of RSV gene expression during infection of A549 cells and MDM. MDM (donor #64) or A549 cells were infected with either NH1125B or NH1067B and at several times post infection, RNA was isolated and viral gene expressions was determined with RNA–SEQ. Reads that did not map to the human genome were used to determine viral gene expression. Hours post infection are designated by the number following the virus (e.g. NH1067-4.0 represents the 4.0 hour time point). The Y-axis represents the magnitude of viral gene expression or total gene reads normalized to housekeeping genes (see Methods).
(PPTX)

S1 Table Host gene expression following infection with RSV strains NH1125B and NH1076. The table represents the number of up-regulated or down-regulated during infection.
(TIF)
