# Supplementary figures and images for: Distinct patterns of innate immune activation by clinical isolates of respiratory syncytial virus
Source: PLoS One. 2017 Sep 6;12(9):e0184318. doi: 10.1371/journal.pone.0184318 (PMC5587315; doi:10.1371/journal.pone.0184318)

## Slide 1
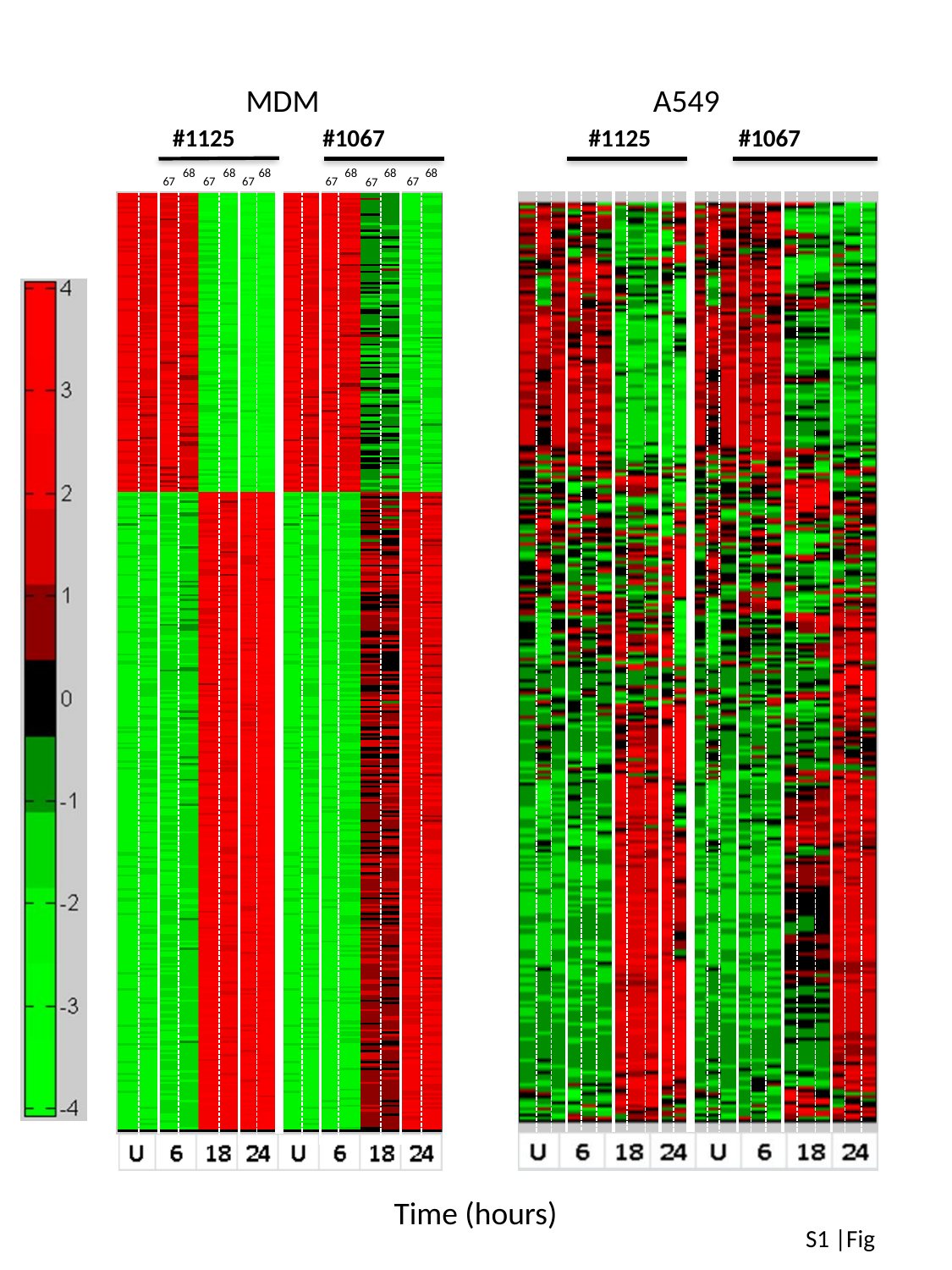

MDM
A549
#1125 #1067
#1125 #1067
68
68
68
68
68
68
67
67
67
67
67
67
Time (hours)
S1 |Fig

Supplement: S1 Fig — A549 cells or MDM (donor #67 and #68) were infected with NH1067B and NH1125B. RNA was extracted at the designated times post infection and gene expression was determined by RNA-SEQ. Individual genes are in rows and variations in expression are depicted using the color scale on the left of the figure. In MDM heatmap, for each time point, the left column represents donor #67 and the right column represents donor #68. For A549 cells, each column at each time point represents a biological replicate. Mock infected (U) cells were used as controls. (PPTX) [file pone.0184318.s001.pptx]

## Slide 1
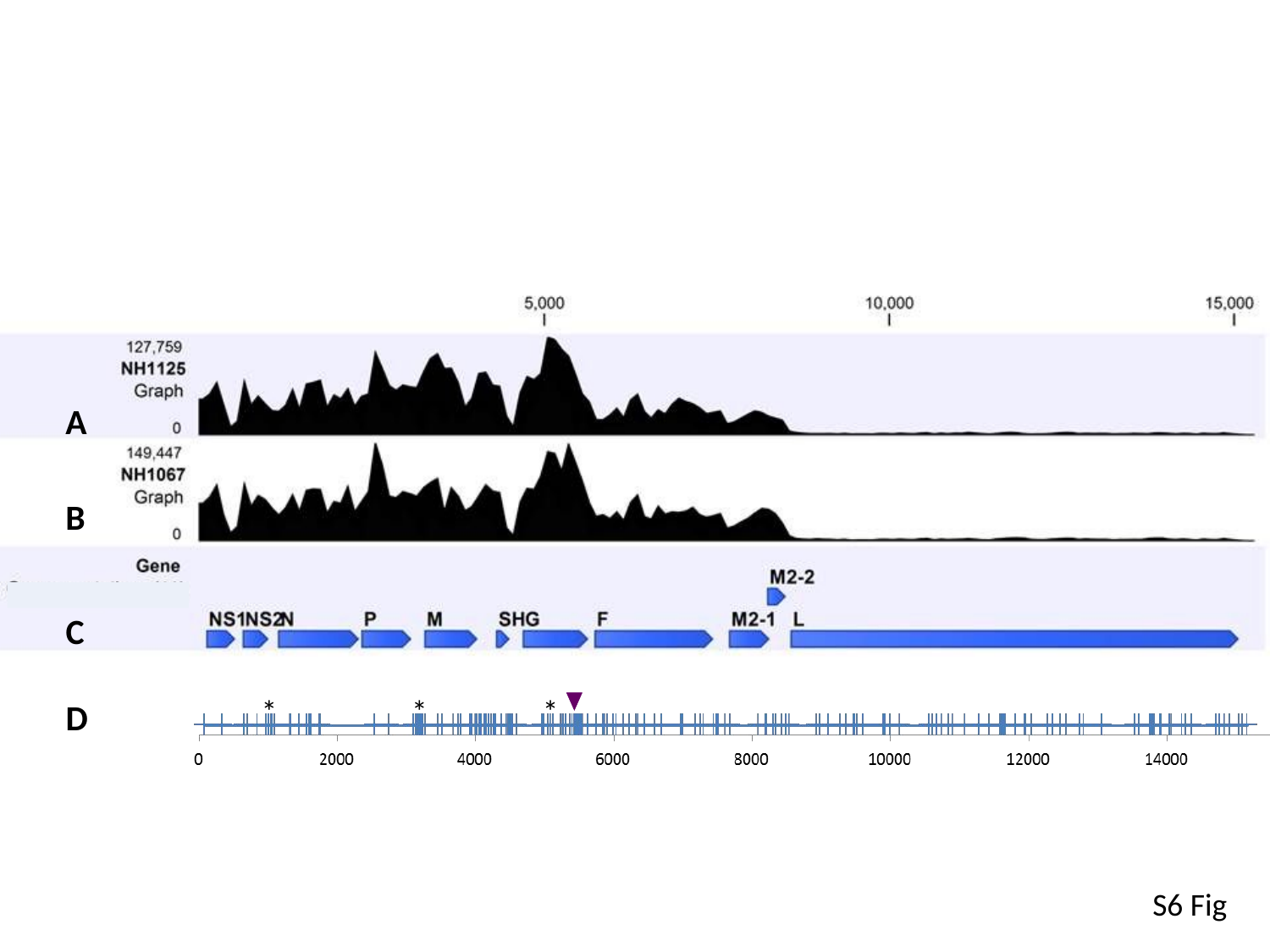

A
B
C
*
*
*
D
S6 Fig

Supplement: S6 Fig — MDM (donor #66) were infected with NH1125B (A) or NH1067B (B) and at 18 hours post infection, RNA was isolated and gene expression was determined by RNA-SEQ. Here, reads that did not map to the human genome were aligned to the viral genome. The Y-axis represents the magnitude of transcription for each region of the viral genome. A map of the viral genome (C) including the coding regions for the RSV genes is displayed beneath the graph of the gene expression. (D) Comparison of the genome sequence of clinical isolates NH1125B and NH1067B. Each vertical bar represents a nucleotide polymorphism between the 2 strains. The "*" represent 1 or 3 base insertions and the triangle designates the location of the 60 base duplication in the G gene of NH1067B. (PPTX) [file pone.0184318.s006.pptx]

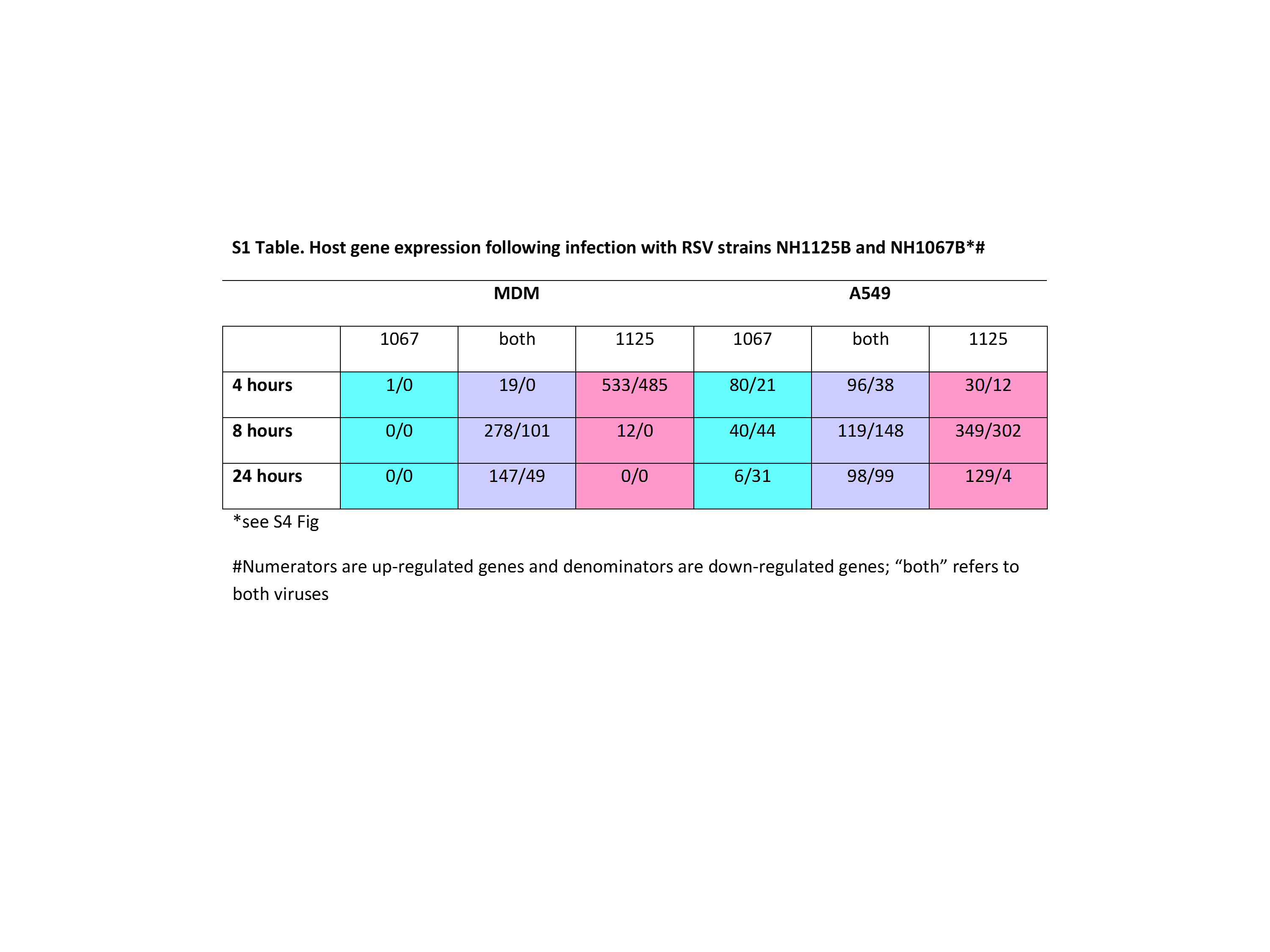

Supplement: S1 Table — The table represents the number of up-regulated or down-regulated during infection. (TIF) [file pone.0184318.s008.tif]
