# Supplementary material for: Distinct patterns of innate immune activation by clinical isolates of respiratory syncytial virus
Source: PLoS One. 2017 Sep 6;12(9):e0184318. doi: 10.1371/journal.pone.0184318 (PMC5587315; doi:10.1371/journal.pone.0184318)
Supplement: S2 Fig — Pathway induced during RSV infection (NH1125B) include A) RIG-I; B) TLR; C) Inflammasome; D) IL-6 signaling; E) NF-ĸB; F) Interferon; G) Death receptor signaling; H) DNA damage response and; I) Cell cycle control of chromosomal replication. Red indicates increased transcription and green indicates decreased transcription (based on RNA-SEQ data). (PPTX) [file pone.0184318.s002.pptx]

## Slide 1
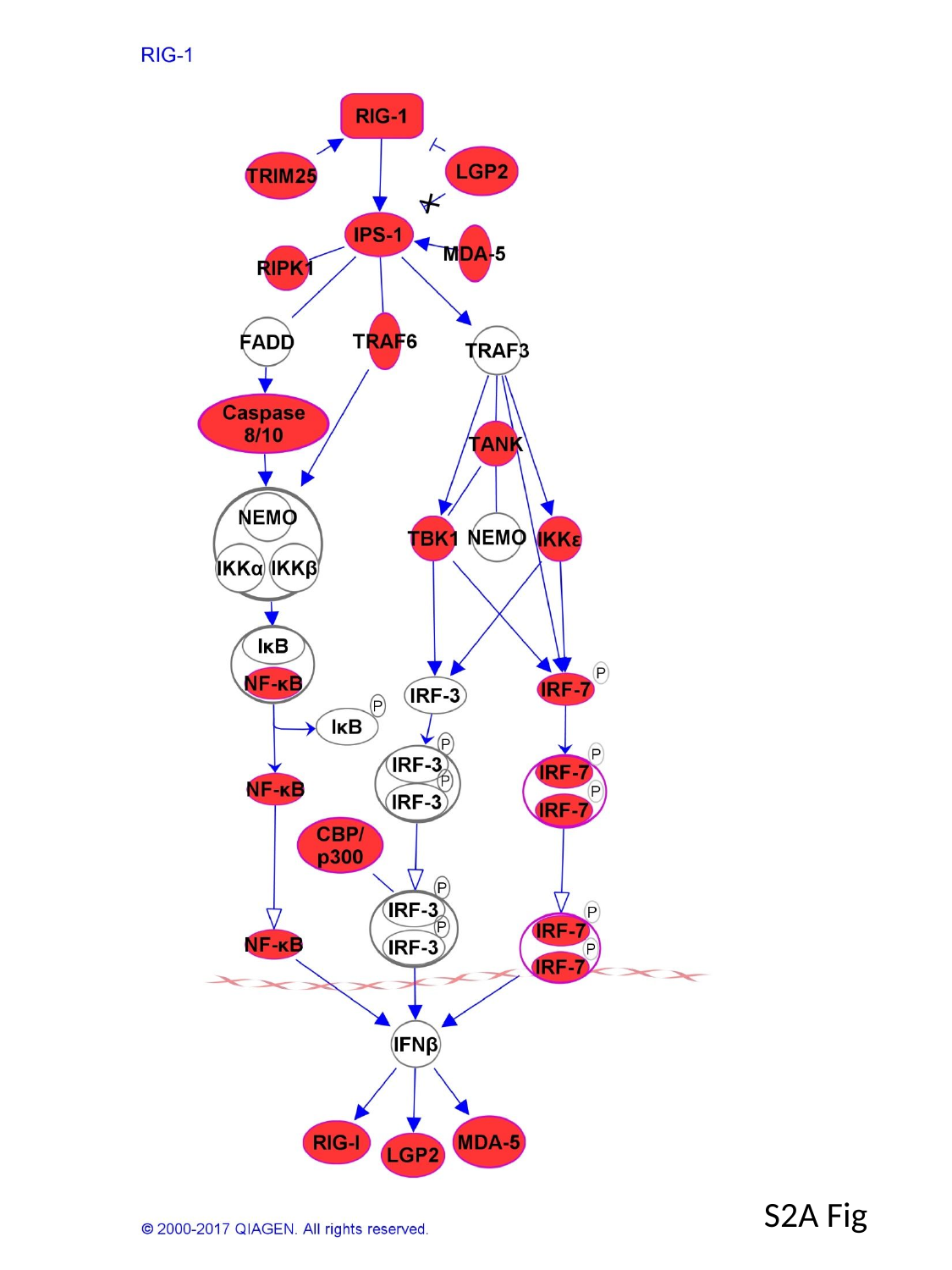

S2A Fig

## Slide 2
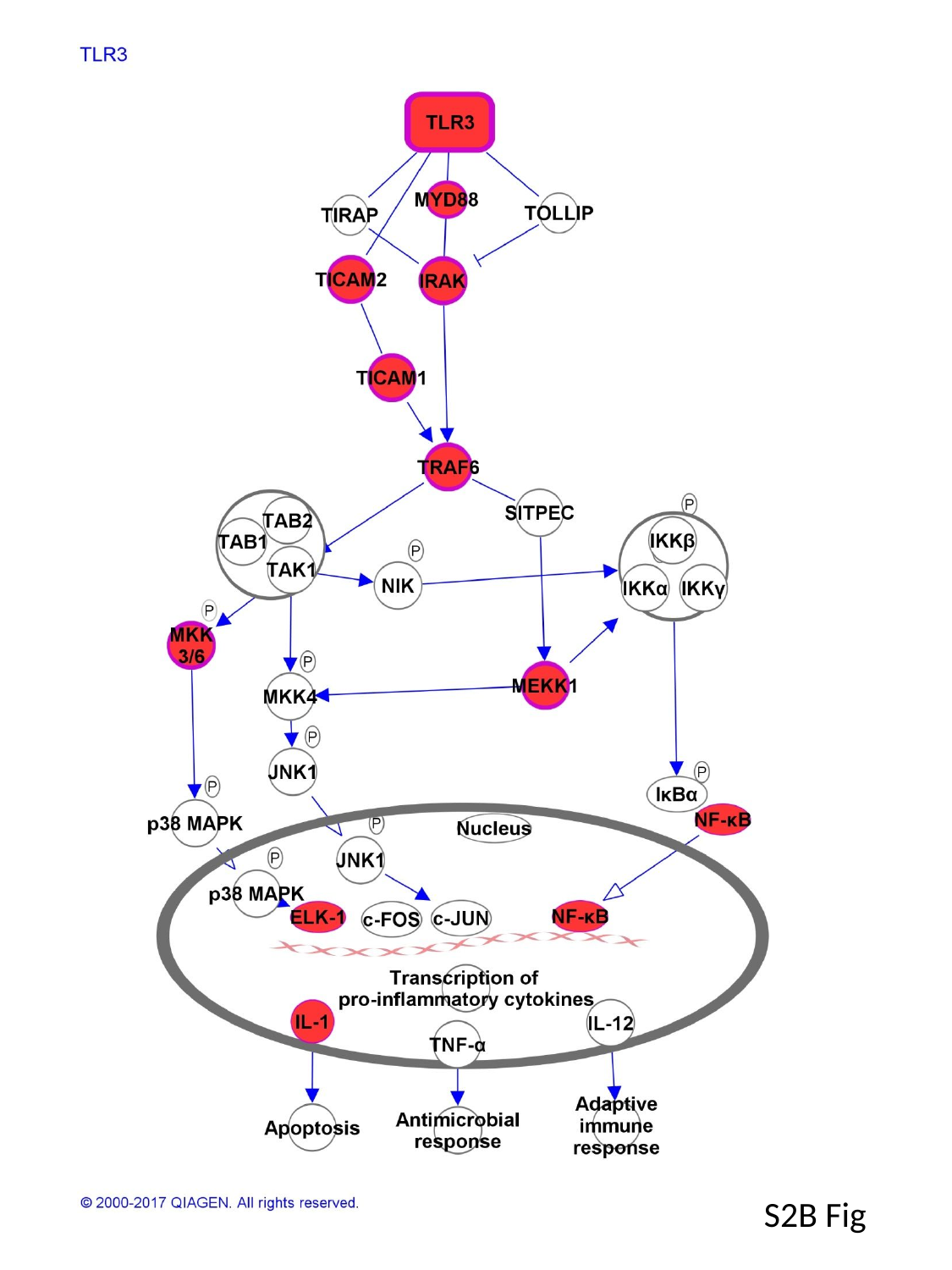

S2B Fig

## Slide 3
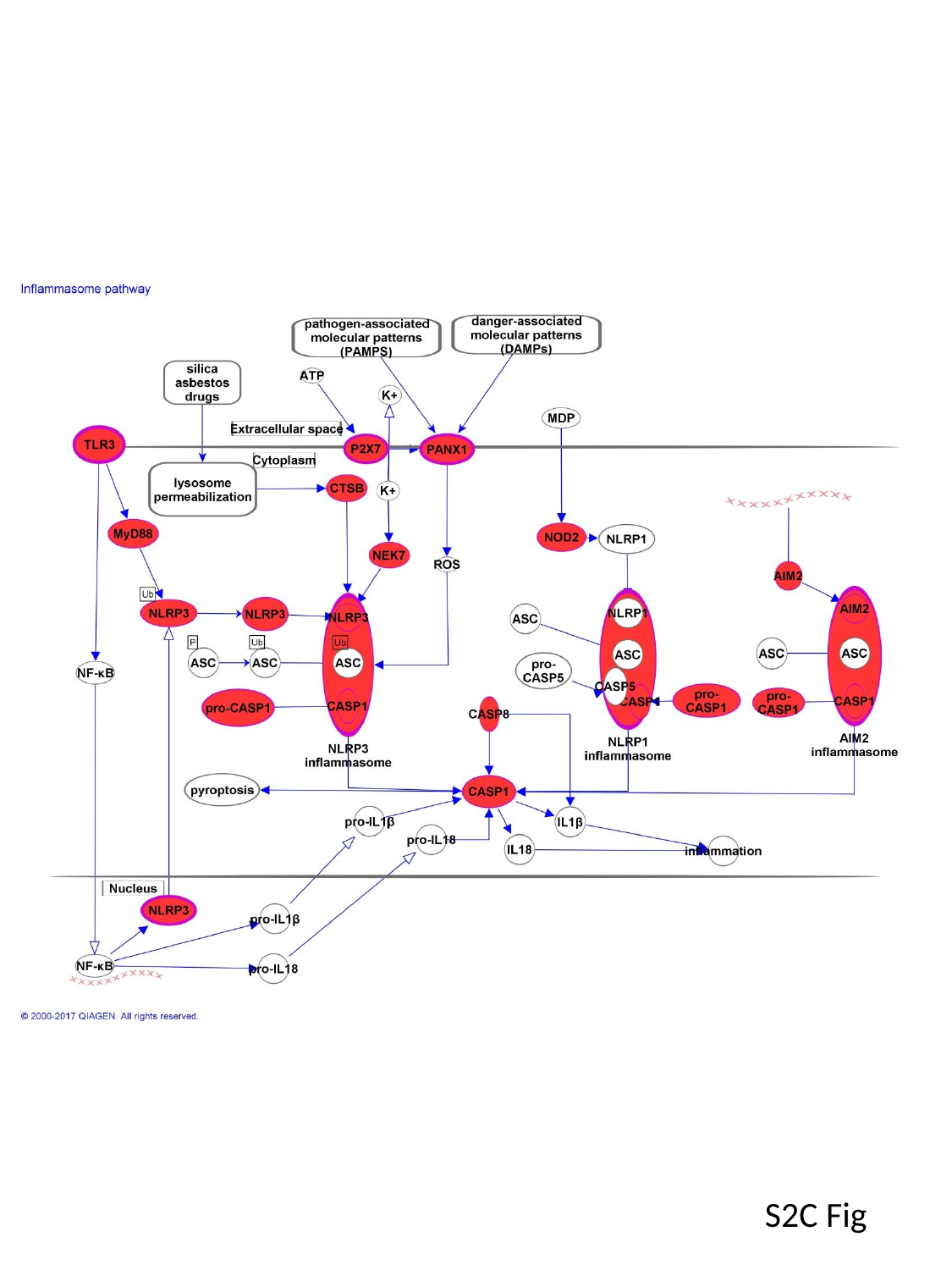

S2C Fig

## Slide 4
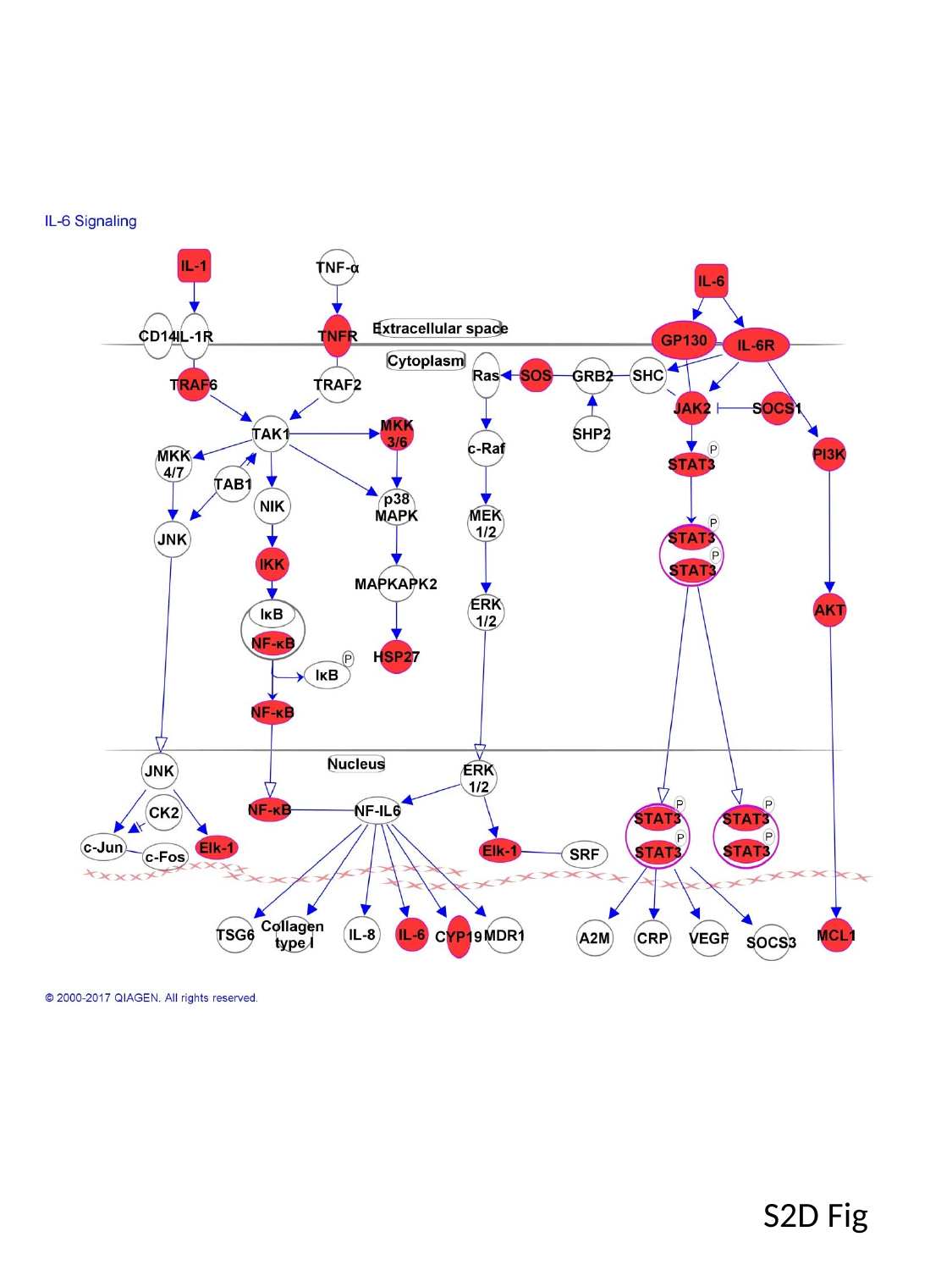

S2D Fig

## Slide 5
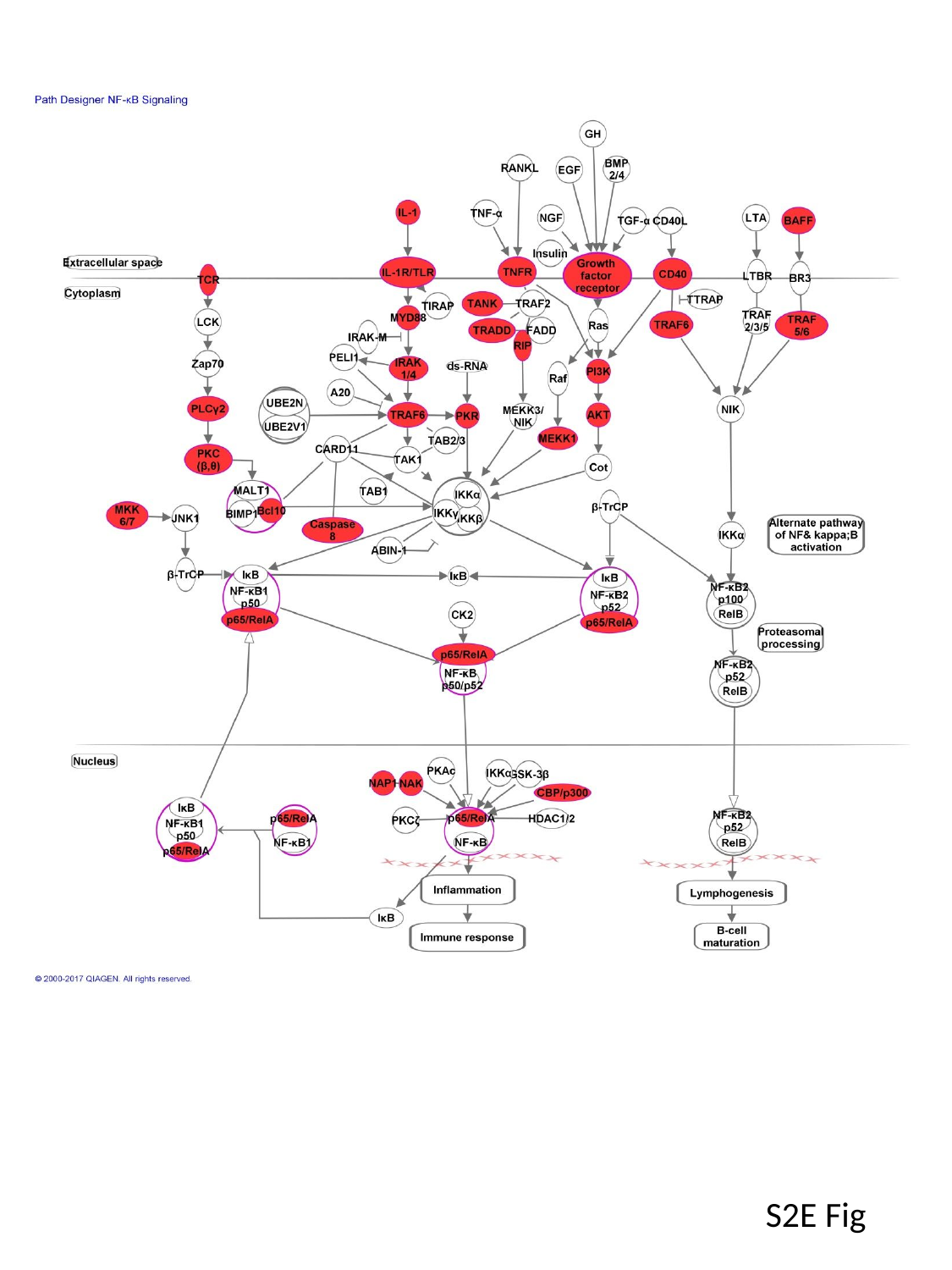

S2E Fig

## Slide 6
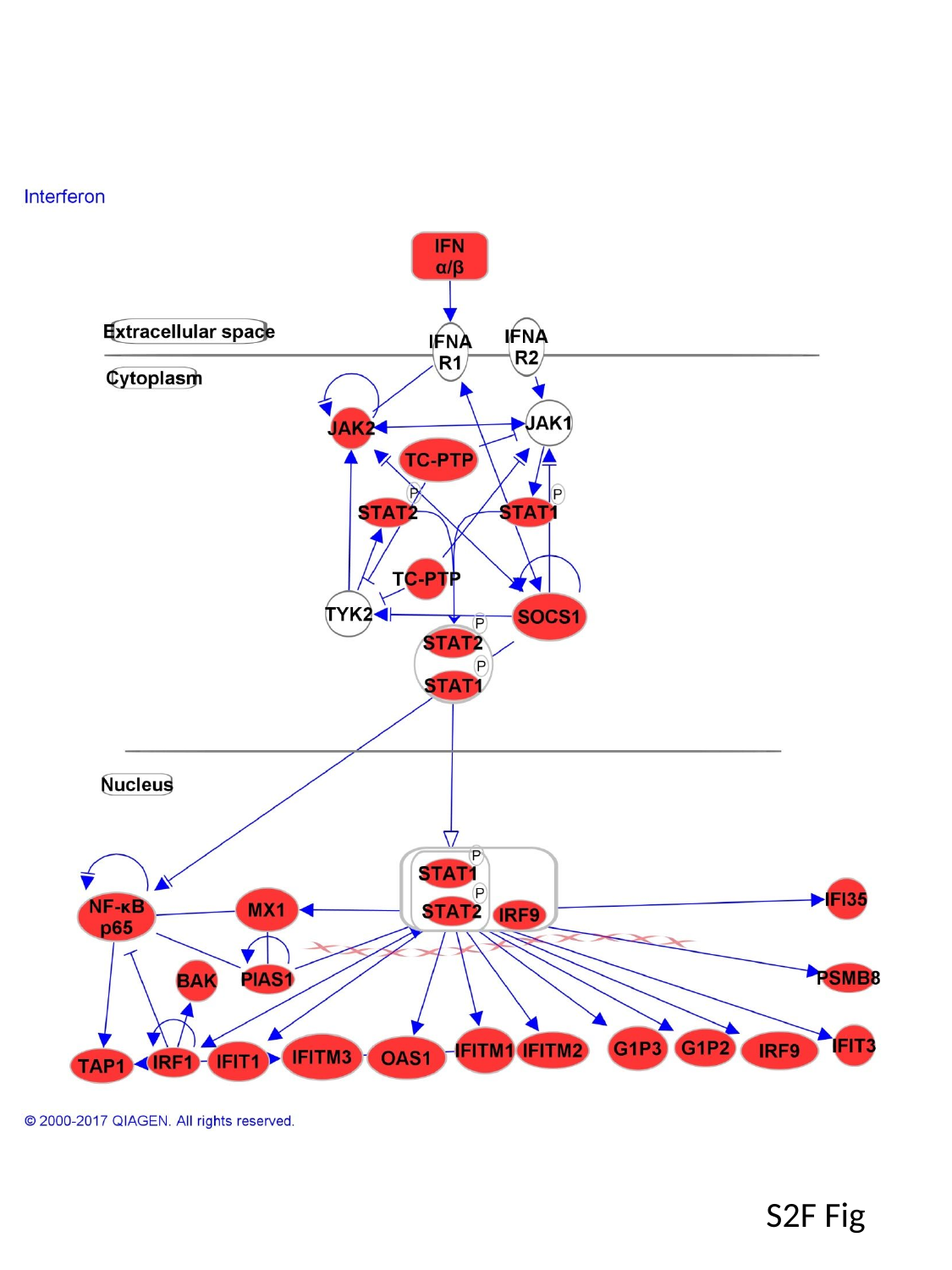

S2F Fig

## Slide 7
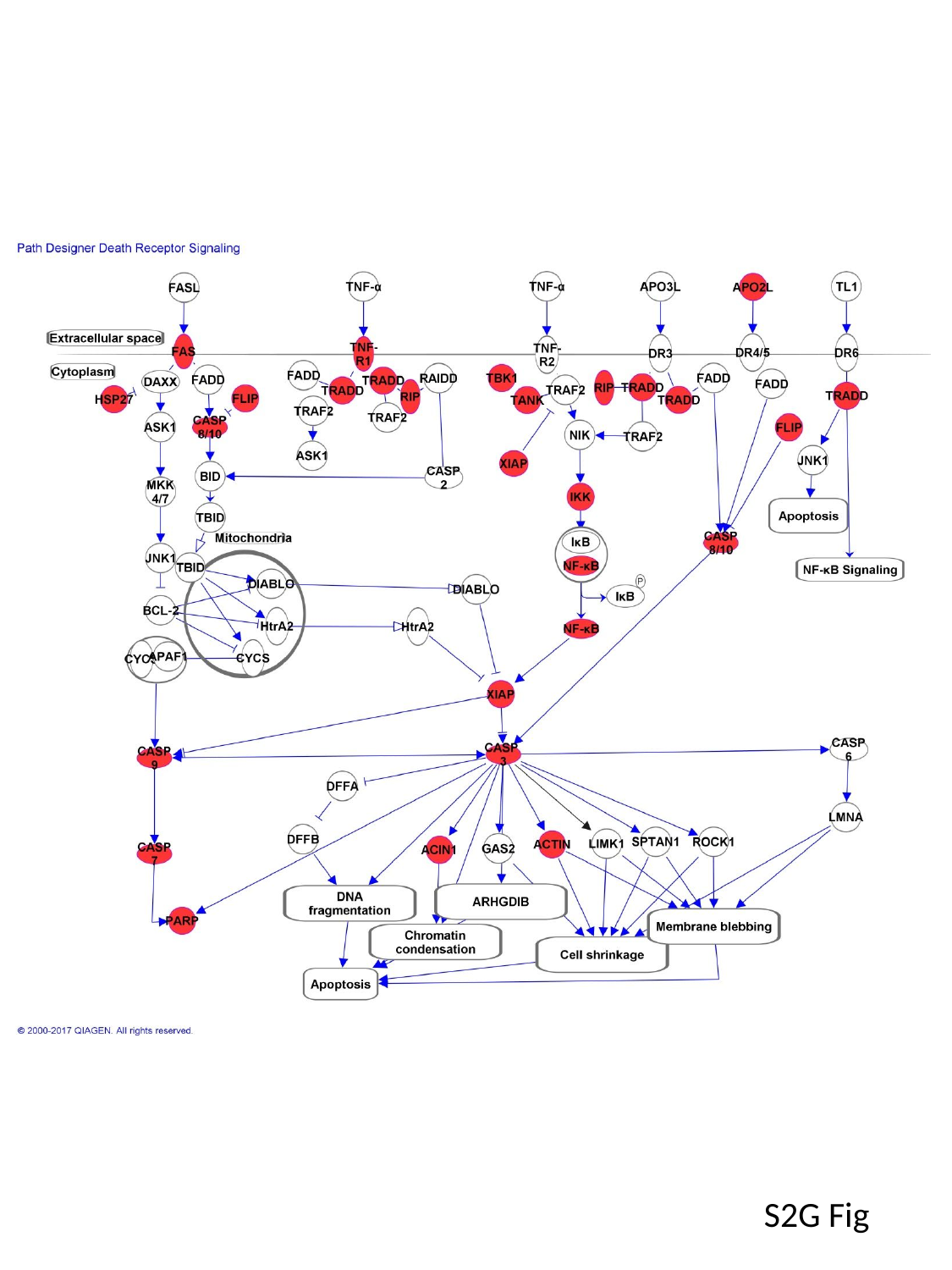

S2G Fig

## Slide 8
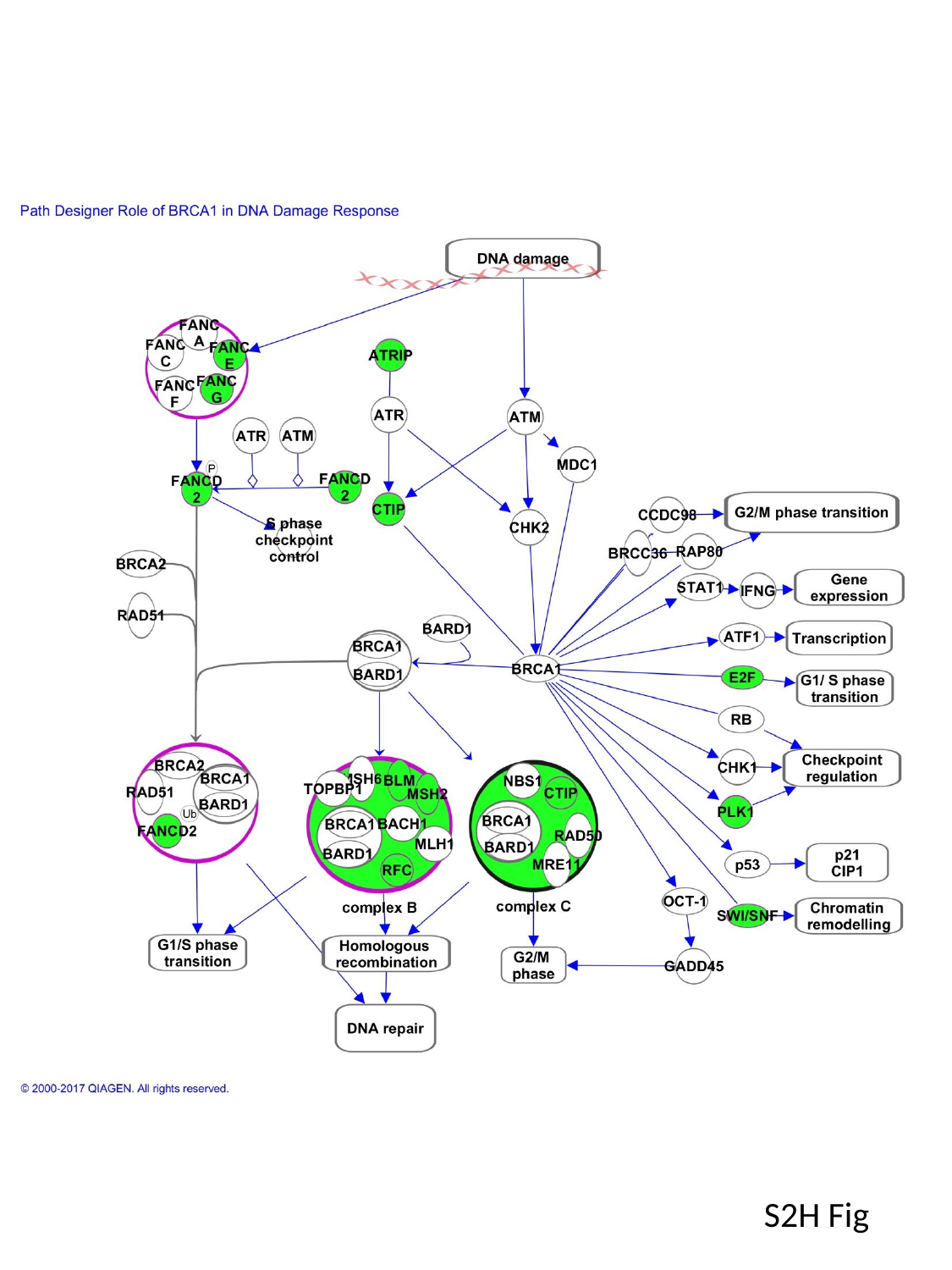

S2H Fig

## Slide 9
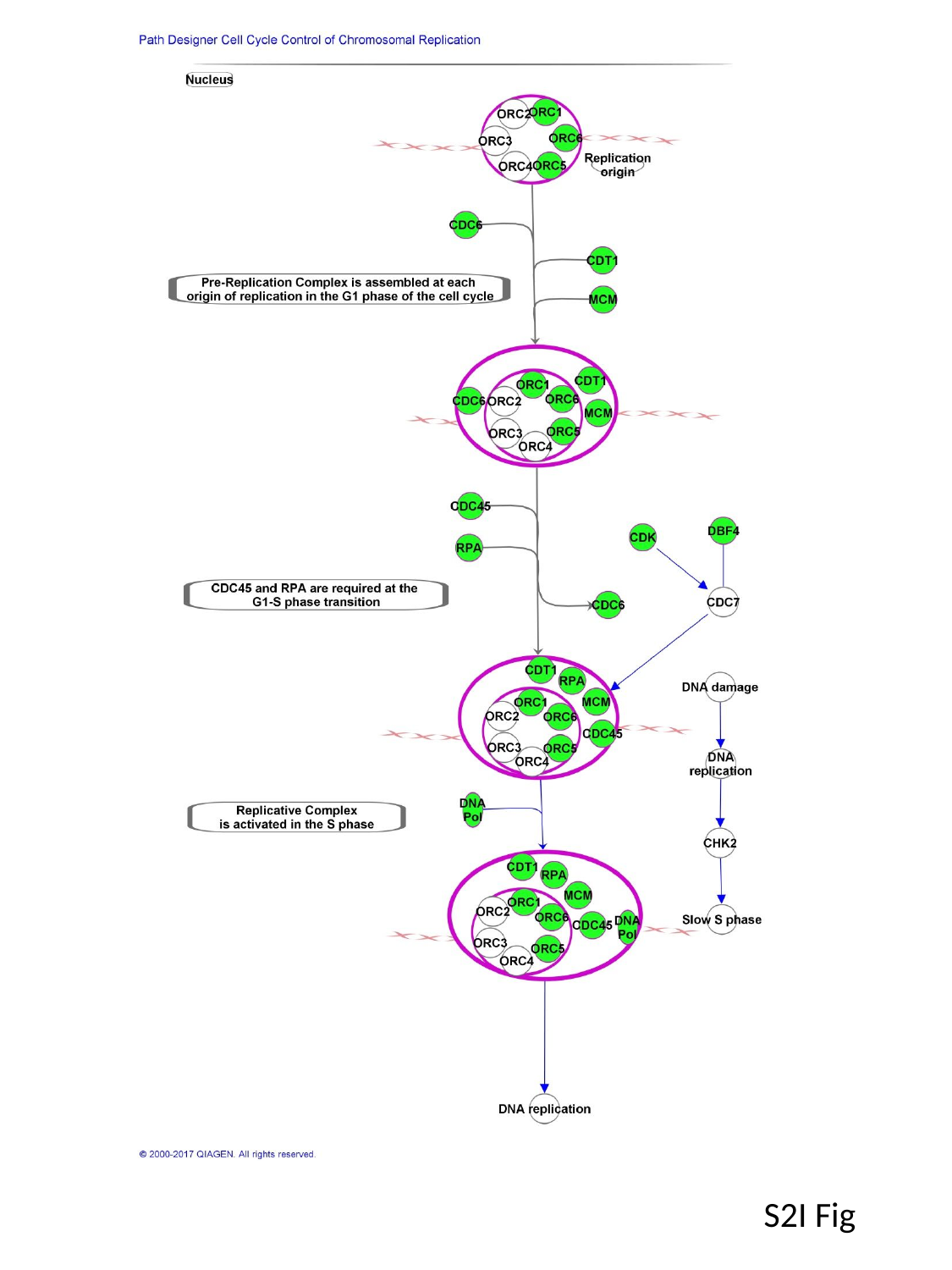

S2I Fig
