# Supplementary material for: Distinct patterns of innate immune activation by clinical isolates of respiratory syncytial virus
Source: PLoS One. 2017 Sep 6;12(9):e0184318. doi: 10.1371/journal.pone.0184318 (PMC5587315; doi:10.1371/journal.pone.0184318)
Supplement: S3 Fig — MDM (donor #96 and #98) were infected with clinical isolate NH1125B (1125) or 1125 pre-treated with an RSV-neutralizing antibody (1125+SYN). As controls, cells were incubated with an RSV-neutralizing antibody (SYN), TLR7 stimulator (R848) or incubated with R848 and SYN (R848+SYN). At 20 hours post-infection, cell culture supernatants were collected and concentration of IL-6 (A, C) and RANTES (B, D) were measured by BioPlex assay. (PPTX) [file pone.0184318.s003.pptx]

## Slide 1
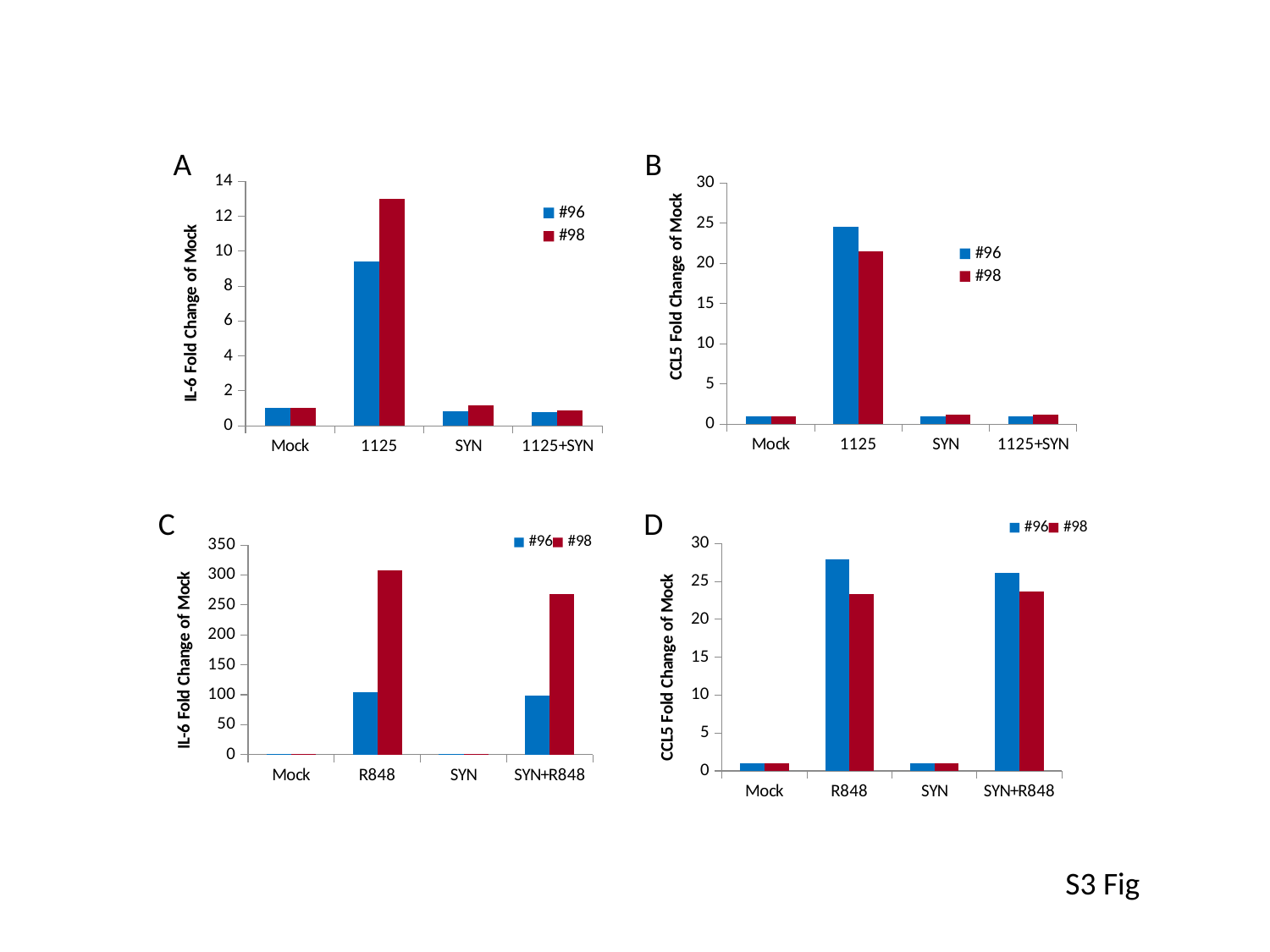

A
B
### Chart
| Category | #96 | #98 |
|---|---|---|
| Mock | 1.0 | 1.0 |
| 1125 | 9.431088082901555 | 13.002266931141968 |
| SYN | 0.8461139896373056 | 1.1663360725417964 |
| 1125+SYN | 0.783160621761658 | 0.8815528478322471 |
### Chart
| Category | #96 | #98 |
|---|---|---|
| Mock | 1.0 | 1.0 |
| 1125 | 24.594306049822062 | 21.553158705701076 |
| SYN | 1.0249110320284698 | 1.197226502311248 |
| 1125+SYN | 1.0 | 1.2234206471494606 |
### Chart
| Category | #96 | #98 |
|---|---|---|
| Mock | 1.0 | 1.0 |
| R848 | 27.87460815047022 | 23.33385093167702 |
| SYN | 0.9874608150470219 | 1.0481366459627328 |
| SYN+R848 | 26.147335423197493 | 23.709627329192546 |
### Chart
| Category | #96 | #98 |
|---|---|---|
| Mock | 1.0 | 1.0 |
| R848 | 104.62958648806057 | 307.4292216659081 |
| SYN | 0.9592312172393709 | 1.215748748293127 |
| SYN+R848 | 98.61211415259173 | 267.59444697314524 |C
D
S3 Fig
