# Supplementary material for: Distinct patterns of innate immune activation by clinical isolates of respiratory syncytial virus
Source: PLoS One. 2017 Sep 6;12(9):e0184318. doi: 10.1371/journal.pone.0184318 (PMC5587315; doi:10.1371/journal.pone.0184318)
Supplement: S4 Fig — MDM (donor #64) or A549 cells were infected with clinical isolate NH1125B (1125) or NH1067B (1067). At several time points post infection, RNA was isolated and gene expression was determined by RNA-SEQ. In the scatter plot diagrams of cellular gene expression in MDM or A549 cells, each circle represents a specific gene (expressed as reads per kilobase per million (RPKM)) relative to expression of that gene in uninfected cells. Gene expression in cells infected with NH1125B (1125) are displayed as red dots and gene expression in cells infected with NH1067B (1067) are displayed as light blue dots. Genes associated with specific pathways are labeled. Venn diagram displays differential cellular gene expression of MDMs infected with clinical isolates NH1125B (pink), NH1067B (blue) or both (purple) at different time point post-infection compared to mock control. The numbers in the diagram, also displayed in the S1 Table, represent up-regulated genes (numerators) and down-regulated genes (denominators). (PPTX) [file pone.0184318.s004.pptx]

## Slide 1
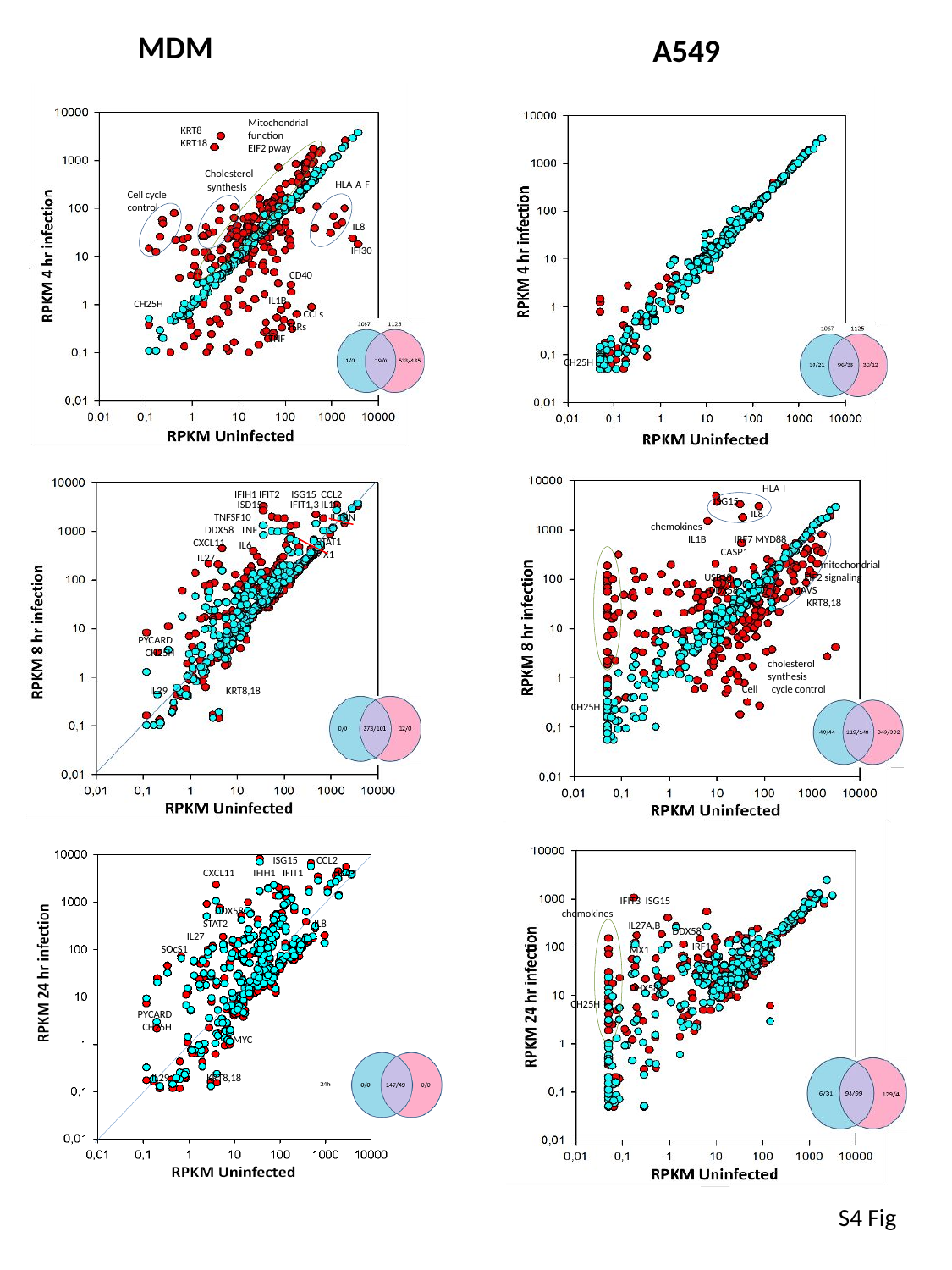

MDM
A549
Mitochondrial
function
EIF2 pway
KRT8
KRT18
Cholesterol
 synthesis
HLA-A-F
Cell cycle
control
IL8
IFI30
 CD40
IL1B
 CCLs
 TLRs
TNF
CH25H
CH25H
 HLA-I
 ISG15
 IL8
 chemokines
 IL1B IRF7 MYD88
 CASP1
 mitochondrial
 USP18 EIF2 signaling
 DDX58 MAVS
 KRT8,18
 IFIH1 IFIT2 ISG15 CCL2
 IL6
 IL27
 ISD15 IFIT1,3 IL1R
 TNFSF10 IL1RN
 DDX58 TNF
 CXCL11
 STAT1
 MX1
 PYCARD
 CH25H
 IL29 KRT8,18
 cholesterol
 synthesis
Cell cycle control
CH25H
 ISG15 CCL2
 CXCL11 IFIH1 IFIT1 HLA-I
 DDX58
 STAT2 IL8
 IL27
 SOcS1
 IFIT3 ISG15
 chemokines
IL27A,B
DDX58
IRF1
MX1
DHX58
CH25H
 PYCARD
 CH25H
 MYC
 IL29 KRT8,18
S4 Fig
