# Supplementary material for: Distinct patterns of innate immune activation by clinical isolates of respiratory syncytial virus
Source: PLoS One. 2017 Sep 6;12(9):e0184318. doi: 10.1371/journal.pone.0184318 (PMC5587315; doi:10.1371/journal.pone.0184318)
Supplement: S5 Fig — (A-E). Functional fingerprint of cellular gene expression during RSV infection. MDM (donor #64) were infected with clinical isolates NH1125B or NH1067B and at several times post-infection, RNA was isolated and cellular gene expression was determined by RNA-SEQ. Genes are clustered based on their functionality which is described on the left side of the figure. The gene expression profiles correspond to the data represented in Figs 3 and 4 and S4 Fig. (PPTX) [file pone.0184318.s005.pptx]

## Slide 1
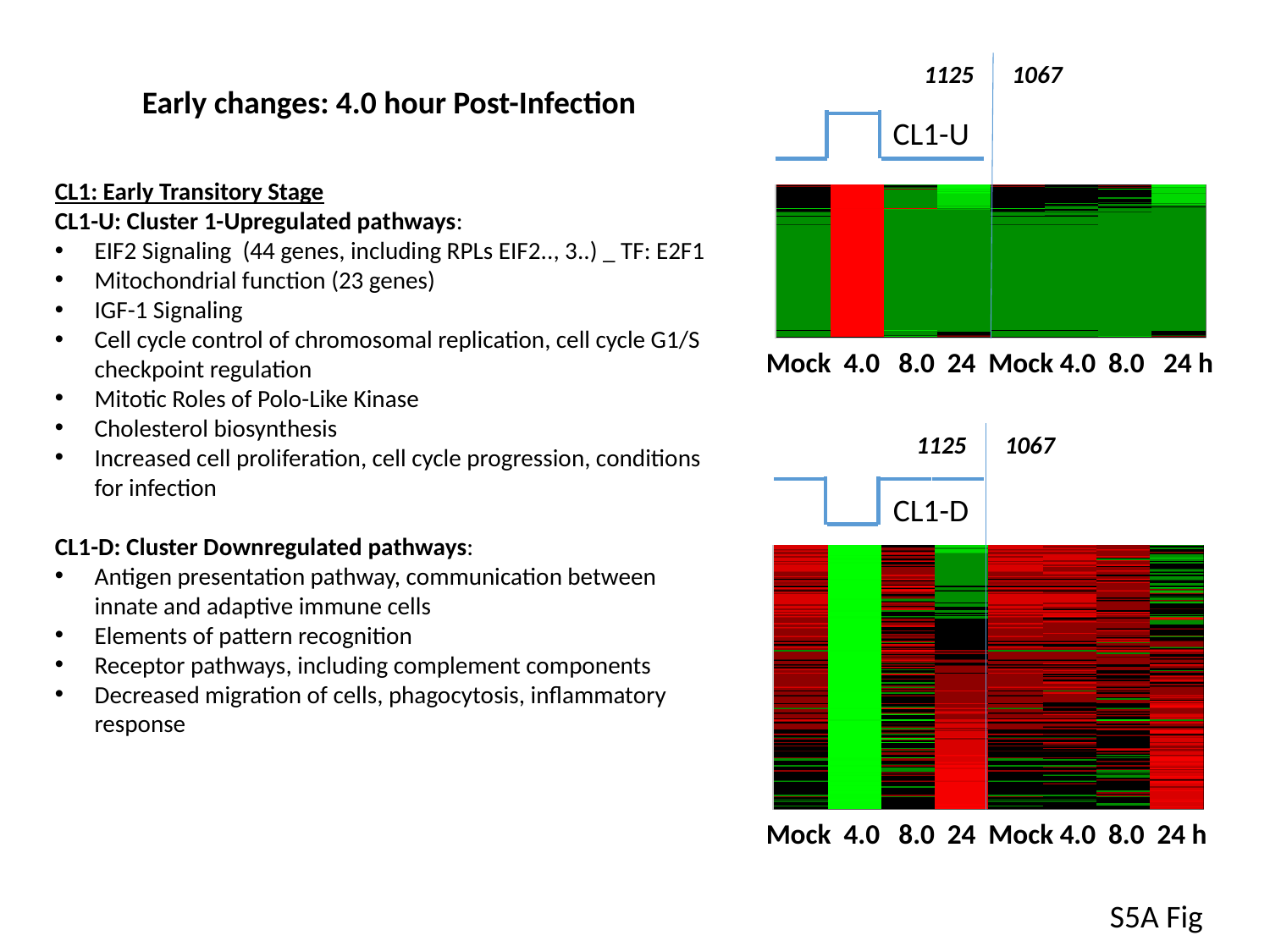

1125 1067
Early changes: 4.0 hour Post-Infection
CL1-U
CL1: Early Transitory Stage
CL1-U: Cluster 1-Upregulated pathways:
EIF2 Signaling (44 genes, including RPLs EIF2.., 3..) _ TF: E2F1
Mitochondrial function (23 genes)
IGF-1 Signaling
Cell cycle control of chromosomal replication, cell cycle G1/S checkpoint regulation
Mitotic Roles of Polo-Like Kinase
Cholesterol biosynthesis
Increased cell proliferation, cell cycle progression, conditions for infection
CL1-D: Cluster Downregulated pathways:
Antigen presentation pathway, communication between innate and adaptive immune cells
Elements of pattern recognition
Receptor pathways, including complement components
Decreased migration of cells, phagocytosis, inflammatory response
 Mock 4.0 8.0 24 Mock 4.0 8.0 24 h
1125 1067
CL1-D
 Mock 4.0 8.0 24 Mock 4.0 8.0 24 h
S5A Fig

## Slide 2
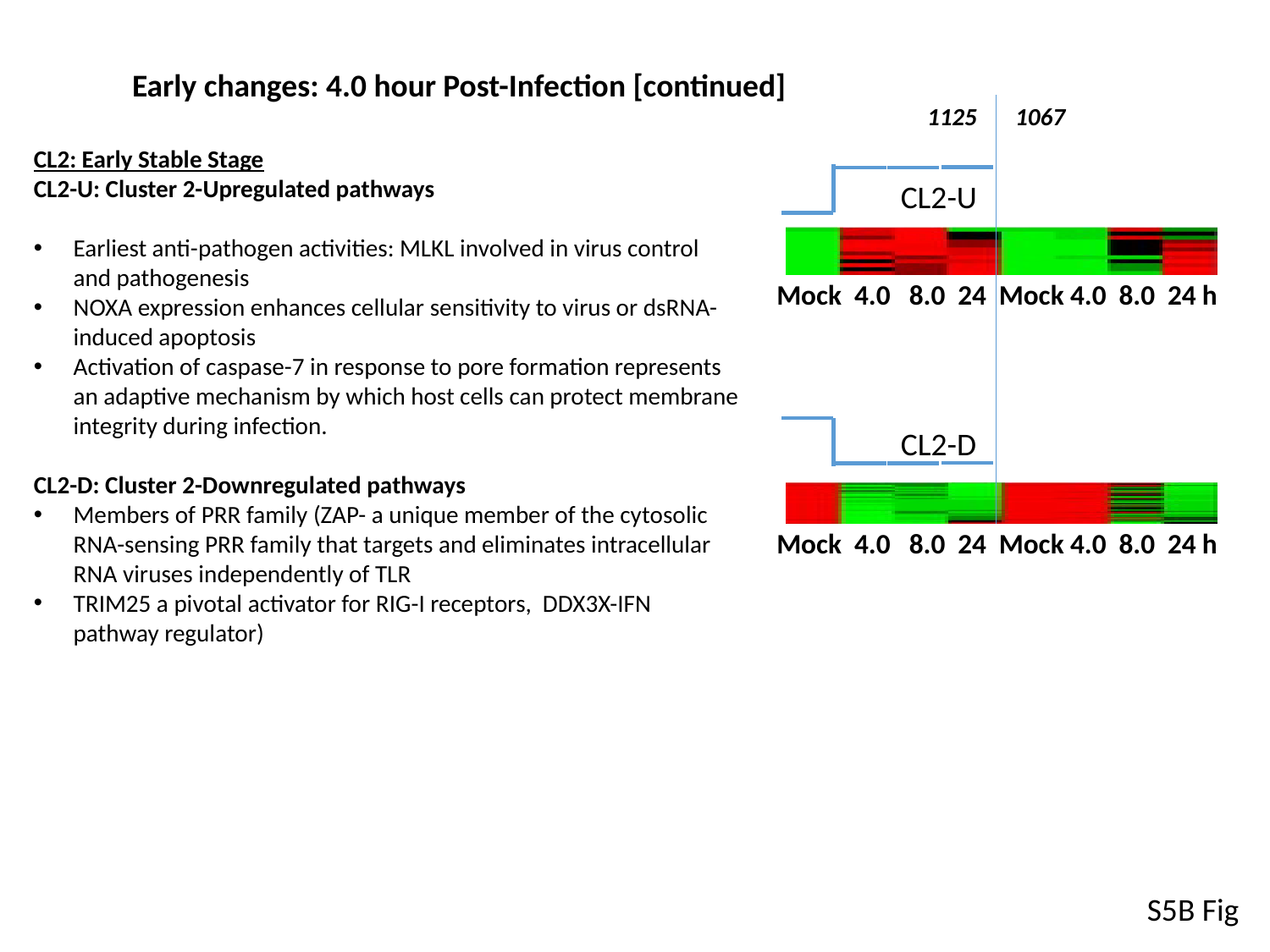

Early changes: 4.0 hour Post-Infection [continued]
1125 1067
CL2: Early Stable Stage
CL2-U: Cluster 2-Upregulated pathways
Earliest anti-pathogen activities: MLKL involved in virus control and pathogenesis
NOXA expression enhances cellular sensitivity to virus or dsRNA-induced apoptosis
Activation of caspase-7 in response to pore formation represents an adaptive mechanism by which host cells can protect membrane integrity during infection.
CL2-D: Cluster 2-Downregulated pathways
Members of PRR family (ZAP- a unique member of the cytosolic RNA-sensing PRR family that targets and eliminates intracellular RNA viruses independently of TLR
TRIM25 a pivotal activator for RIG-I receptors, DDX3X-IFN pathway regulator)
CL2-U
 Mock 4.0 8.0 24 Mock 4.0 8.0 24 h
CL2-D
 Mock 4.0 8.0 24 Mock 4.0 8.0 24 h
S5B Fig

## Slide 3
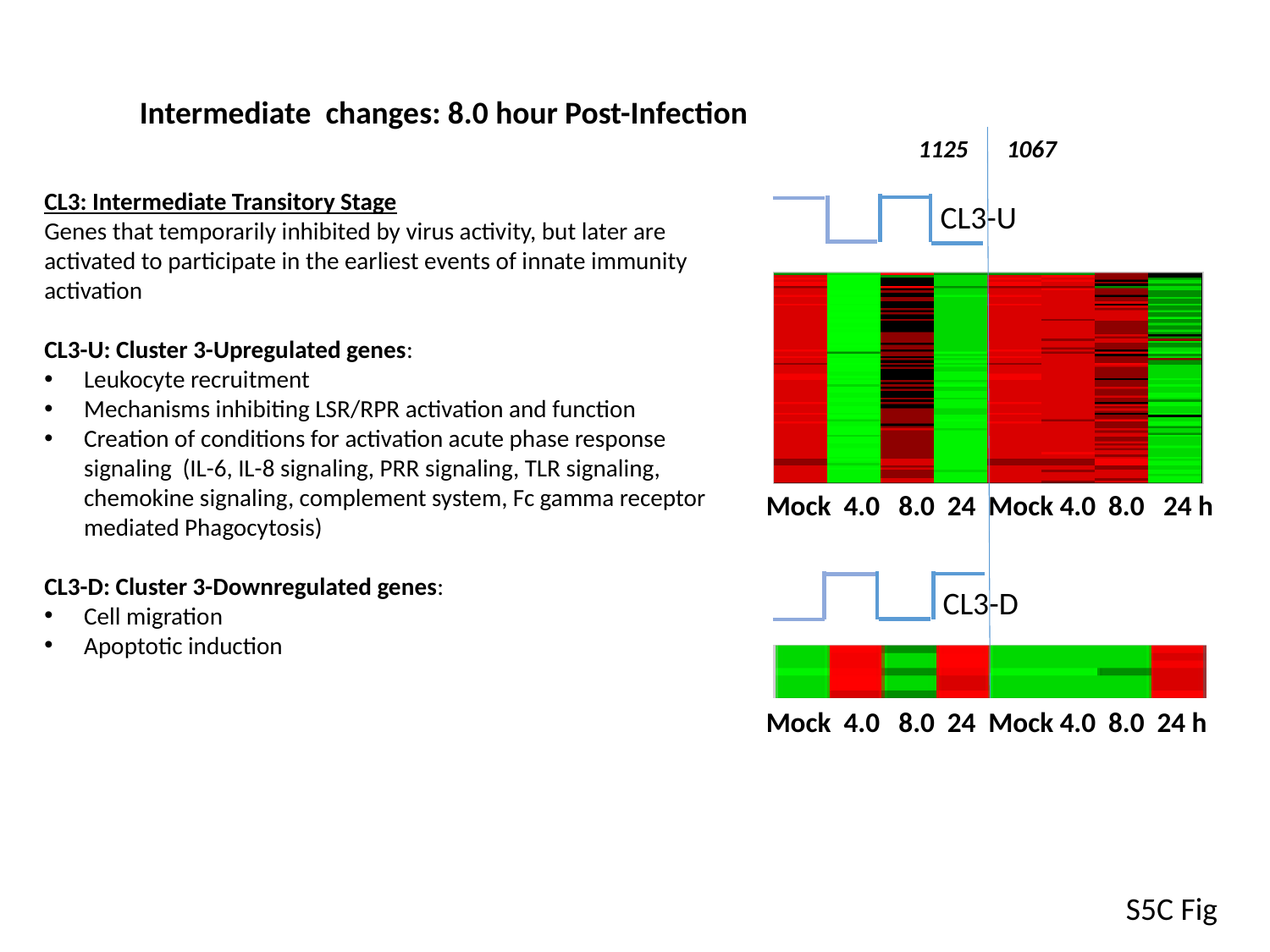

Intermediate changes: 8.0 hour Post-Infection
1125 1067
CL3: Intermediate Transitory Stage
Genes that temporarily inhibited by virus activity, but later are activated to participate in the earliest events of innate immunity activation
CL3-U: Cluster 3-Upregulated genes:
Leukocyte recruitment
Mechanisms inhibiting LSR/RPR activation and function
Creation of conditions for activation acute phase response signaling (IL-6, IL-8 signaling, PRR signaling, TLR signaling, chemokine signaling, complement system, Fc gamma receptor mediated Phagocytosis)
CL3-D: Cluster 3-Downregulated genes:
Cell migration
Apoptotic induction
CL3-U
 Mock 4.0 8.0 24 Mock 4.0 8.0 24 h
CL3-D
 Mock 4.0 8.0 24 Mock 4.0 8.0 24 h
S5C Fig

## Slide 4
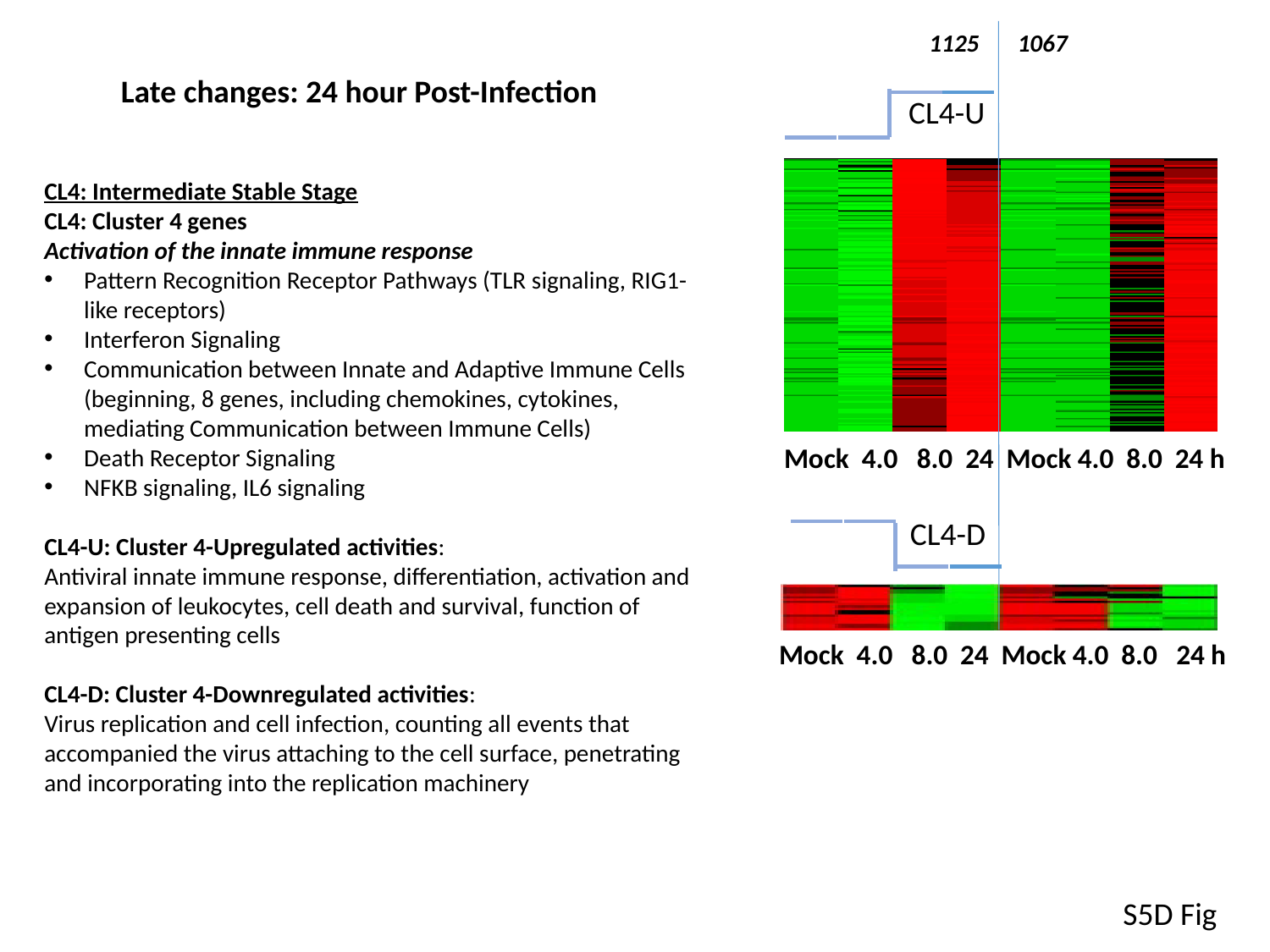

1125 1067
Late changes: 24 hour Post-Infection
CL4-U
CL4: Intermediate Stable Stage
CL4: Cluster 4 genes
Activation of the innate immune response
Pattern Recognition Receptor Pathways (TLR signaling, RIG1-like receptors)
Interferon Signaling
Communication between Innate and Adaptive Immune Cells (beginning, 8 genes, including chemokines, cytokines, mediating Communication between Immune Cells)
Death Receptor Signaling
NFKB signaling, IL6 signaling
CL4-U: Cluster 4-Upregulated activities:
Antiviral innate immune response, differentiation, activation and expansion of leukocytes, cell death and survival, function of antigen presenting cells
CL4-D: Cluster 4-Downregulated activities:
Virus replication and cell infection, counting all events that accompanied the virus attaching to the cell surface, penetrating and incorporating into the replication machinery
 Mock 4.0 8.0 24 Mock 4.0 8.0 24 h
CL4-D
 Mock 4.0 8.0 24 Mock 4.0 8.0 24 h
S5D Fig

## Slide 5
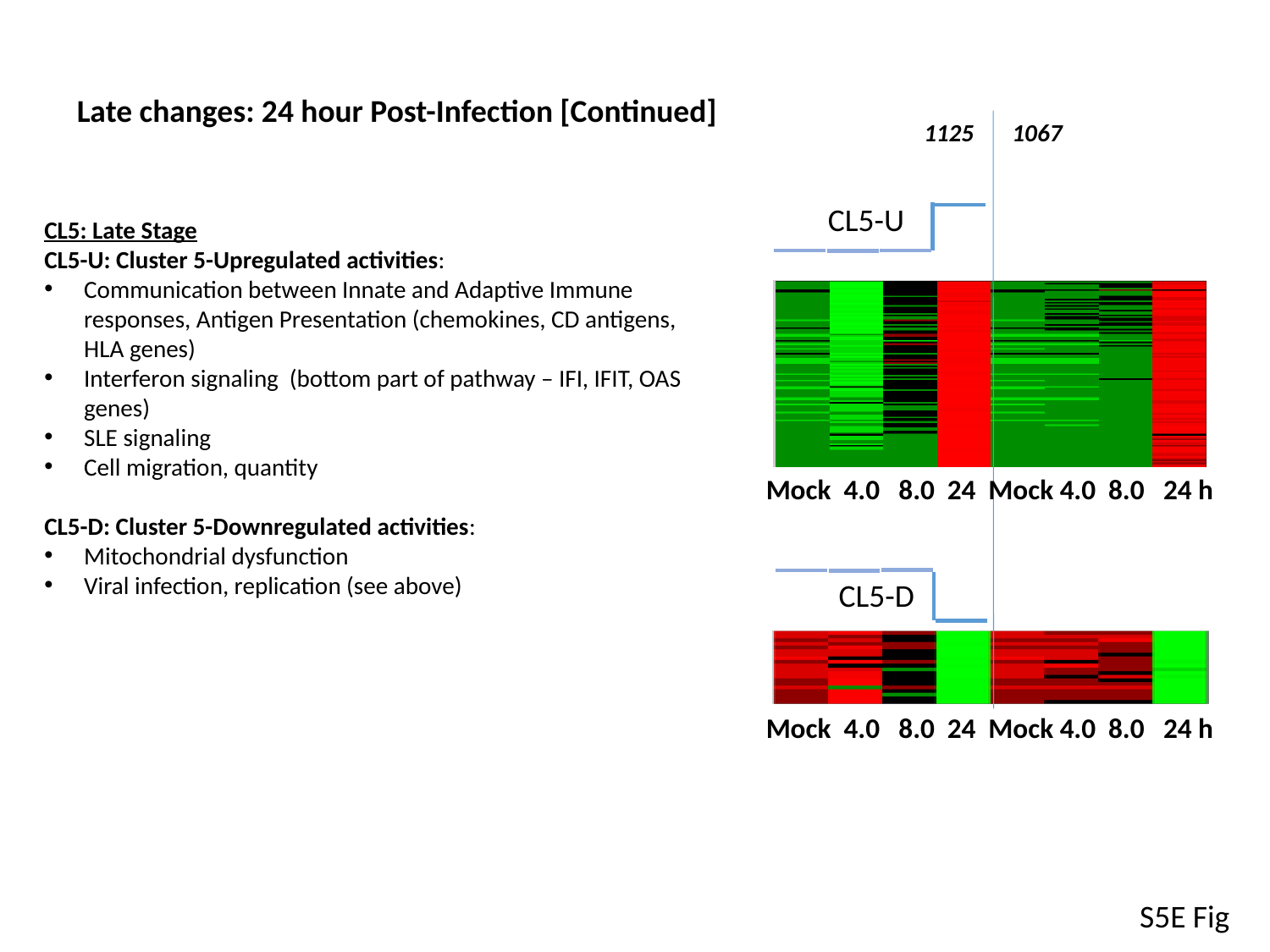

Late changes: 24 hour Post-Infection [Continued]
1125 1067
CL5: Late Stage
CL5-U: Cluster 5-Upregulated activities:
Communication between Innate and Adaptive Immune responses, Antigen Presentation (chemokines, CD antigens, HLA genes)
Interferon signaling (bottom part of pathway – IFI, IFIT, OAS genes)
SLE signaling
Cell migration, quantity
CL5-D: Cluster 5-Downregulated activities:
Mitochondrial dysfunction
Viral infection, replication (see above)
CL5-U
 Mock 4.0 8.0 24 Mock 4.0 8.0 24 h
CL5-D
 Mock 4.0 8.0 24 Mock 4.0 8.0 24 h
S5E Fig
