# Supplementary material for: Distinct patterns of innate immune activation by clinical isolates of respiratory syncytial virus
Source: PLoS One. 2017 Sep 6;12(9):e0184318. doi: 10.1371/journal.pone.0184318 (PMC5587315; doi:10.1371/journal.pone.0184318)
Supplement: S7 Fig — MDM (donor #64) or A549 cells were infected with either NH1125B or NH1067B and at several times post infection, RNA was isolated and viral gene expressions was determined with RNA–SEQ. Reads that did not map to the human genome were used to determine viral gene expression. Hours post infection are designated by the number following the virus (e.g. NH1067-4.0 represents the 4.0 hour time point). The Y-axis represents the magnitude of viral gene expression or total gene reads normalized to housekeeping genes (see Methods). (PPTX) [file pone.0184318.s007.pptx]

## Slide 1
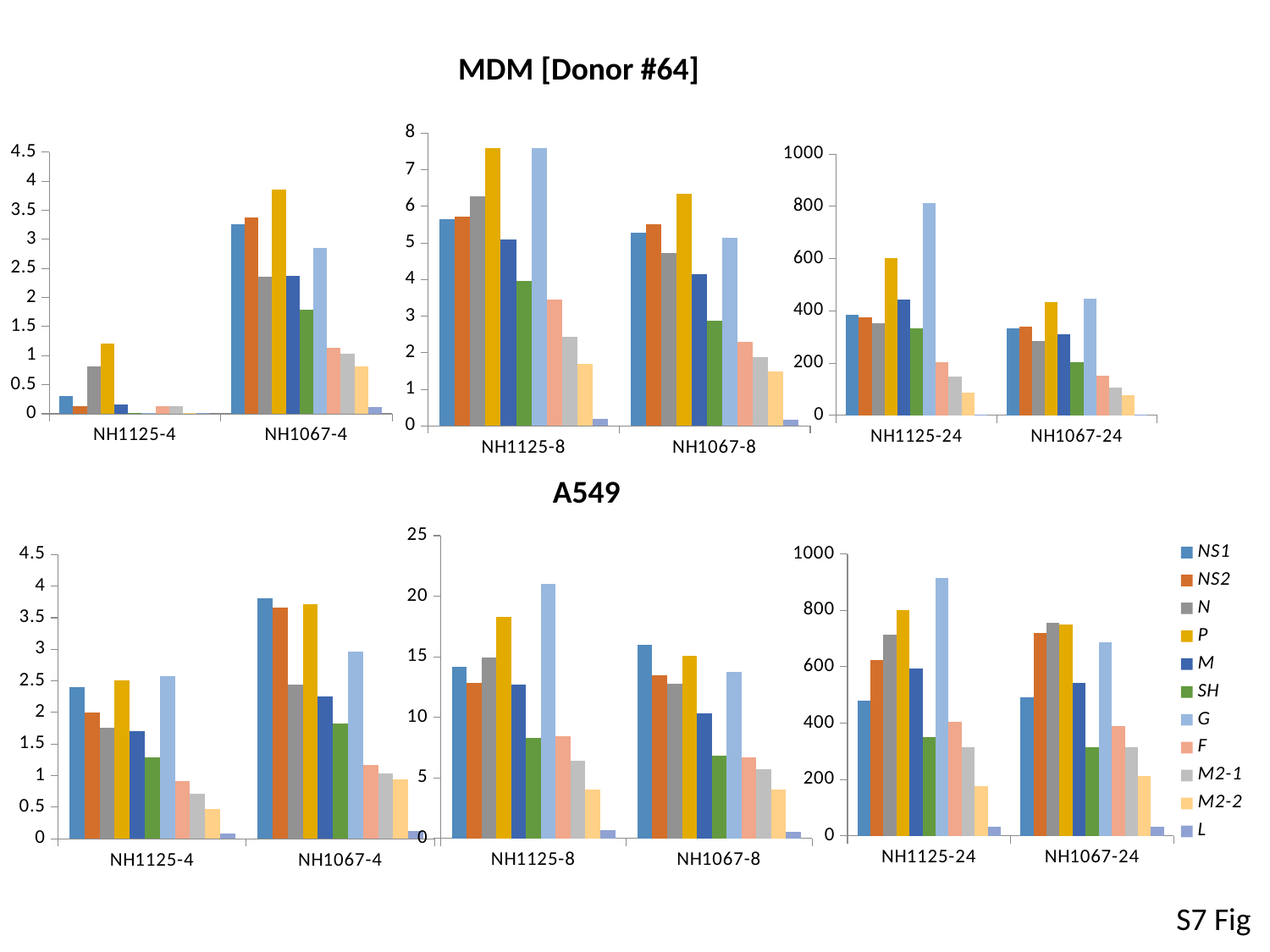

MDM [Donor #64]
### Chart
| Category | NS1 | NS2 | N | P | M | SH | G | F | M2-1 | M2-2 | L |
|---|---|---|---|---|---|---|---|---|---|---|---|
| NH1125-4 | 0.30047601566244186 | 0.12573765578489876 | 0.8089738883219589 | 1.2072526503411525 | 0.1618847136164009 | 0.00700410292919445 | 0.02030243349070553 | 0.12863187292572761 | 0.12500179615470502 | 0.01523969648330221 | 0.01685251161643741 |
| NH1067-4 | 3.256890055739401 | 3.3769138288402947 | 2.355710062588049 | 3.8634193118264384 | 2.368209408516178 | 1.7950958665860233 | 2.8532559374869613 | 1.1373727410689043 | 1.0379631580758193 | 0.810921172007526 | 0.11778119942618083 |
### Chart
| Category | NS1 | NS2 | N | P | M | SH | G | F | M2-1 | M2-2 | L |
|---|---|---|---|---|---|---|---|---|---|---|---|
| NH1125-24 | 386.2591958553891 | 375.4708334970111 | 351.24794847273 | 600.7515002921349 | 442.22764208169247 | 332.71528294997034 | 813.1109968726645 | 204.3248377203449 | 148.29323482579835 | 88.07475311732136 | 2.578095104297736 |
| NH1067-24 | 333.996867658034 | 338.94537356844546 | 285.2442769296465 | 434.45136946884026 | 309.98444524875816 | 202.529329504762 | 446.96895725663614 | 151.7632610629094 | 107.591465856941 | 77.34555672259843 | 2.608115179191397 |
### Chart
| Category | NS1 | NS2 | N | P | M | SH | G | F | M2-1 | M2-2 | L |
|---|---|---|---|---|---|---|---|---|---|---|---|
| NH1125-8 | 5.648788637849731 | 5.7292904442979715 | 6.26621437268447 | 7.584554569290453 | 5.106656230890776 | 3.9569386229898753 | 7.595153145247106 | 3.4435106922194656 | 2.4321694600176915 | 1.6936260746039624 | 0.19027932493193608 |
| NH1067-8 | 5.276816410832693 | 5.512568331710511 | 4.736708985422503 | 6.346940018623939 | 4.15701310784545 | 2.8782634968178327 | 5.148513327997111 | 2.2911619187957974 | 1.8704947340125935 | 1.4916038949978465 | 0.1667841870081087 |A549
### Chart
| Category | NS1 | NS2 | N | P | M | SH | G | F | M2-1 | M2-2 | L |
|---|---|---|---|---|---|---|---|---|---|---|---|
| NH1125-8 | 14.18671629165953 | 12.83988337360111 | 14.955055670594753 | 18.26637460017638 | 12.717229130640069 | 8.2811423171037 | 20.988257033508336 | 8.410373566335888 | 6.422677492206507 | 4.045599727745033 | 0.6423160345683888 |
| NH1067-8 | 15.988593305475701 | 13.49356762120365 | 12.756819505338541 | 15.106884073059412 | 10.308220485450471 | 6.815961966469816 | 13.742206124533295 | 6.681007037821156 | 5.674701704051743 | 4.04429759841689 | 0.5435013212148366 |
### Chart
| Category | NS1 | NS2 | N | P | M | SH | G | F | M2-1 | M2-2 | L |
|---|---|---|---|---|---|---|---|---|---|---|---|
| NH1125-24 | 480.48756780494716 | 623.1260943657082 | 715.0925577873936 | 799.8096939222814 | 595.0568935455681 | 350.9307103387229 | 916.4217919029983 | 405.2284246519389 | 314.13280884839884 | 175.01594626783395 | 33.397443994938186 |
| NH1067-24 | 490.33704669350277 | 719.060776058817 | 754.5749528046183 | 748.2512359780658 | 541.8129332618757 | 313.86762643687126 | 687.7428836441543 | 389.60975524040316 | 315.139538000101 | 212.23991258198328 | 32.592648590222666 |
### Chart
| Category | NS1 | NS2 | N | P | M | SH | G | F | M2-1 | M2-2 | L |
|---|---|---|---|---|---|---|---|---|---|---|---|
| NH1125-4 | 2.4067771772137836 | 2.004548662665727 | 1.7578153132432235 | 2.5095004870529776 | 1.6972262701635499 | 1.2938050828110628 | 2.5774168771162054 | 0.9162668086212333 | 0.7165830846331227 | 0.46737054924067156 | 0.07926689314362899 |
| NH1067-4 | 3.8116600318210643 | 3.6547501653016545 | 2.4440711930989 | 3.7139810674003537 | 2.2591937440394365 | 1.8212877596804666 | 2.9589518671690187 | 1.1686635588707417 | 1.0397840922957104 | 0.9328428642633403 | 0.11707157079889491 |S7 Fig
